# Fine pore engineering in a series of isoreticular metal-organic frameworks for efficient C_2_H_2_/CO_2_ separation

**DOI:** 10.1038/s41467-021-27929-7

**Published:** 2022-01-11

**Authors:** Jun Wang, Yan Zhang, Yun Su, Xing Liu, Peixin Zhang, Rui-Biao Lin, Shixia Chen, Qiang Deng, Zheling Zeng, Shuguang Deng, Banglin Chen

**Affiliations:** 1 School of Resource, Environmental and Chemical Engineering, Nanchang University, Nanchang, 330031 Jiangxi PR China; 2 Jiangxi University of Chinese Medicine, Nanchang, 330031 Jiangxi PR China; 3 Key Laboratory of Biomass Chemical Engineering of Ministry of Education, College of Chemical and Biological Engineering, Zhejiang University, Hangzhou, 310027 Zhejiang PR China; 4 MOE Key Laboratory of Bioinorganic and Synthetic Chemistry, School of Chemistry, Sun Yat-Sen University, Guangzhou, 510006 Guangdong China; 5 School for Engineering of Matter, Transport and Energy, Arizona State University, 551 E. Tyler Mall, Tempe, AZ 85287 USA; 6 Department of Chemistry, University of Texas at San Antonio, One UTSA Circle, San Antonio, TX 78249-0698 USA

**Keywords:** Solid-state chemistry, Porous materials

## Abstract

The separation of C_2_H_2_/CO_2_ is not only industrially important for acetylene purification but also scientifically challenging owing to their high similarities in physical properties and molecular sizes. Ultramicroporous metal-organic frameworks (MOFs) can exhibit a pore confinement effect to differentiate gas molecules of similar size. Herein, we report the fine-tuning of pore sizes in sub-nanometer scale on a series of isoreticular MOFs that can realize highly efficient C_2_H_2_/CO_2_ separation. The subtle structural differences lead to remarkable adsorption performances enhancement. Among four MOF analogs, by integrating appropriate pore size and specific binding sites, [Cu(dps)_2_(SiF_6_)] (SIFSIX-dps-Cu, SIFSIX = SiF_6_^2-^, dps = 4.4’-dipyridylsulfide, also termed as NCU-100) exhibits the highest C_2_H_2_ uptake capacity and C_2_H_2_/CO_2_ selectivity. At room temperature, the pore space of SIFSIX-dps-Cu significantly inhibits CO_2_ molecules but takes up a large amount of C_2_H_2_ (4.57 mmol g^−1^), resulting in a high IAST selectivity of 1787 for C_2_H_2_/CO_2_ separation. The multiple host-guest interactions for C_2_H_2_ in both inter- and intralayer cavities are further revealed by dispersion-corrected density functional theory and grand canonical Monte Carlo simulations. Dynamic breakthrough experiments show a clean C_2_H_2_/CO_2_ separation with a high C_2_H_2_ working capacity of 2.48 mmol g^−1^.

## Abstract (web summary)

The separation of acetylene and carbon dioxide by porous materials requires delicate control over the pore size. Herein, the authors fine-tune the pore size at sub-nanometer scale in a series of isoreticular metal-organic frameworks to control the acetylene/carbon dioxide separation performance; subtle structural differences lead to remarkable performance enhancement.

## Introduction

Acetylene (C_2_H_2_) is a major raw feedstock for the production of various essential polymers and chemicals^1–3^. In industry, C_2_H_2_ is produced by partial CH_4_ combustion and thermal hydrocarbon cracking, in which carbon dioxide (CO_2_) is a worth-noting impurity that can show great impact upon the subsequent industrial processes^4,5^. Currently, energy-intensive solvent extraction and cryogenic distillations are employed to separate C_2_H_2_/CO_2_ mixtures^6^. Due to the close boiling points (189.3 K for C_2_H_2_; 194.7 K for CO_2_), these approaches suffer from low energy efficiency and are environmentally unfriendly^7–9^. Therefore, it is urgent to develop an energy-efficient approach to realize the challenging C_2_H_2_/CO_2_ separation. Adsorption-based gas separation using porous materials represents a promising alternative technology^10–12^. Nevertheless, C_2_H_2_ and CO_2_ gas molecules show identical molecular shapes (dimensions: 3.32 × 3.34 × 5.7  Å^3^ for C_2_H_2_; 3.18 × 3.33 × 5.36 Å^3^ for CO_2_) and kinetic diameters (3.3 Å, Supplementary Fig. 1), making it very challenging to develop high-performance adsorbents for C_2_H_2_/CO_2_ separation through physisorption^13–15^.

Metal–organic frameworks (MOFs) are well-known for their readily tunable pore sizes/shapes and internal surface modification^16–19^. By virtue of the isoreticular principle and building blocks approach in MOF chemistry, the pore adjustment of porous materials has been performed in a more predictable and more precise way^20–24^. By substituting organic linkers and/or metal nodes, the pore space in MOFs can integrate shape matching and specific binding toward targeted gas molecules^25,26^. SIFSIX-type MOFs featuring anionic MF_6_^2−^ groups (M = Si, Ti, Ge, etc.) have been demonstrated as efficient adsorbents for many separations, mainly attributed to their high-density fluorinated sites and high-sieving pore^27^. The fluoride atoms can serve as hydrogen bonding acceptors forming strong interactions with C_2_H_2_^28^. On the other hand, the length of dipyridine linkers that defines the pore aperture is variable upon substitution, thus tuning the aperture size of one-dimensional (1D) pore channels in these MOF materials^29^. This uniqueness makes SIFSIX-type MOFs a prominent platform with several progresses for separation such as C_2_H_2_/C_2_H_4_^30^, C_3_H_4_/C_3_H_6_^31^, C4 isomers separation^32^, and CO_2_ capture^33^. Among these SIFSIX-type materials, a flexible MOF [Zn(dps)_2_(SiF_6_)] (UTSA-300-Zn, SIFSIX-dps-Zn) was able to completely differentiate C_2_H_2_ and CO_2_ gas molecules. However, flexible MOFs usually show negligible gas uptake before gate-opening, which might lead to capture leakage when applied to the breakthrough separation of gas mixture^34–38^. In this context, flexible-robust MOFs with permanent small pores as well as specific binding sites have been employed to selective take up targeted gas molecules, whereas minimizing the co-adsorption of counterpart gases by tuning the gate-opening pressure, which has been demonstrated by [Cu(dps)_2_(SiF_6_)] (SIFSIX-dps-Cu) for size-exclusive adsorption of C_2_H_2_ from C_2_H_4_^39^. To achieve simultaneously high capacity and separation selectivity for more challenging C_2_H_2_/CO_2_ separation, a systematical study on fine-tuning of pore structure in prototypal [Zn(dps)_2_(SiF_6_)] would be a rational approach.

Herein, we demonstrate precise control over pore structure through altering anionic linkers and metal nodes in [Zn(dps)_2_(SiF_6_)] to increase both C_2_H_2_ uptake capacity and C_2_H_2_/CO_2_ selectivity. Combining different anionic linkers, three flexible-robust MOFs with precise modulation of pore cavity sizes in a sub-nanometer scale have been utilized for the challenging C_2_H_2_/CO_2_ separation. By integrating suitable pore size and fluorinated binding sites, the exclusive C_2_H_2_ sorption behavior was retained in [Cu(dps)_2_(SiF_6_)] (SIFSIX-dps-Cu, SIFSIX = SiF_6_^2^^−^, dps = 4.4′-dipyridylsulfide, also termed as NCU-100) with a C_2_H_2_ uptake of 4.57 mmol g^−1^ and negligible CO_2_ uptake, resulting in a high IAST selectivity of 1787 for C_2_H_2_/CO_2_ separation. The highly efficient C_2_H_2_/CO_2_ separation in [Cu(dps)_2_(SiF_6_)] has been validated by molecular modeling studies and dynamic breakthrough experiments.

## Results

### Synthesis and characterization

**Fig. 1:**
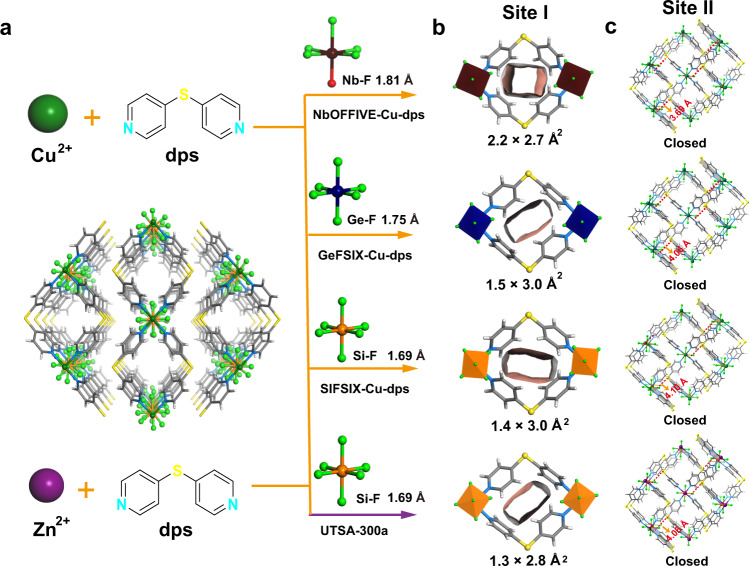
Crystallographic structures. **a** Synthesis procedure of three isoreticular MOFs. Views of **b** Site I and **c** Site II in activated NbOFFIVE-dps-Cu, GeFSIX-dps-Cu, SIFSIX-dps-Cu, and UTSA-300a with varying pore aperture size. Color code: Cu, green; F, light green; S, bright yellow; N, light blue; C, gray; Si, orange; Ge, navy blue; Nb, wine red; and solvent molecules are omitted for clarity.

A series of SIFSIX-dps-Zn variants, SIFSIX-dps-Cu (SIFSIX = SiF_6_^2−^, dps = 4.4′-dipyridylsulfide, termed as NCU-100), GeFSIX-dps-Cu (GeFSIX = GeF_6_^2−^), and NbOFFIVE-dps-Cu (NbOFFIVE = NbOF_5_^2−^) were successfully prepared through solution reactions (Fig. 1a, see Supplementary Information for synthetic and crystallographic details). Crystal structures of the as-synthesized MOFs were determined by single-crystal X-ray diffraction studies, and the phase purities of as-synthesized and activated samples were confirmed by the XRD measurements (Supplementary Figs. 2–4 and Supplementary Table 1). Each Cu(II) atom connects four independent pyridinyl rings of dps ligands and affords 1D chains, generating the intralayer cavity (Site I) with the size of 3.0 × 3.2 Å^2^, 2.5 × 3.1 Å^2^, and 2.3 × 3.1 Å^2^ on NbOFFIVE-dps-Cu, GeFSIX-Cu-dps-Cu, and SIFSIX-dps-Cu, respectively (Fig. 1b and Supplementary Fig. 5a). These apertures are larger than those of the intralayer channels (2.2 × 3.1 Å^2^) in UTSA-300 (SIFSIX-dps-Zn). The chains are further bridged by different anion pillars in the perpendicular direction at Cu(II) sites to form 2D MOF layers containing 1D wavy interlayer channels. The 2D MOF layer planes stack with each other via multiple hydrogen bonds between guest water molecules and F atoms of anion pillars, rendering the structural flexibility and dynamics (Supplementary Fig. 6). The size of the interlayer cavity (Site II) on NbOFFIVE-dps-Cu, GeFSIX-Cu-dps-Cu, and SIFSIX-dps-Cu are 3.2 × 4.8 Å^2^, 2.8 × 4.8 Å^2^, and 2.9 × 4.4 Å^2^, respectively (Fig. 1c and Supplementary Fig. 5b). These results illustrate that both interlayer and intralayer cavities can be finely tuned by altering the anion pillars due to different M–F distances (1.69 Å for Si···F, 1.75 Å for Ge···F, and 1.81 Å for Nb···F).

Although the as-synthesized samples have a similar layered stacking pattern, notable structural transformations are observed after activation, and the activated crystal structures are determined by Rietveld refinements (Fig. 1 and Supplementary Fig. 7 and Supplementary Tables 2–5). In contrast to the Zn analog UTSA-300 (Site I: 1.3 × 2.8 Å^2^ vs. 2.3 × 3.1 Å^2^ for as-synthesized), all Cu-based dynamic layered MOFs displayed slightly expanded intralayer cavity owing to the elongated Cu–F bonds (2.27 Å) than Zn–F bond (2.09 Å). As shown in Fig. 1, the activated NbOFFIVE-dps-Cu showed the largest pore aperture size of 2.2 × 2.7 Å^2^ at Site I, in contrast to those in GeFSIX-dps-Cu (1.5 × 3.0 Å^2^) and SIFSIX-dps-Cu (1.4 × 3.0 Å^2^). The expanded pore spaces are conducive for potential C_2_H_2_ diffusion. Furthermore, the removal of solvent allows the interlayer π–π stacking of dps ligands, giving closed interlayer cavities (Supplementary Fig. 8). The interlayer distance (S atoms to the 2D layer center) was measured to be 4.10 Å in SIFSIX-dps-Cu, and followed by GeFSIX-dps-Cu (4.06 Å), UTSA-300 (4.05 Å), and NbOFFIVE-dps-Cu (3.69 Å), leading to varying interlayer pore spaces upon different packing density^13,40^. The changes of powder X-ray diffraction (PXRD) patterns and corresponding structural transformation in the three isoreticular MOFs upon desolvation or C_2_H_2_-loading are the same as the prototypical zinc analogue UTSA-300 (Supplementary Figs. 2–4 and Supplementary Tables 2–5)^13^.

### Adsorption and separation performances

**Fig. 2:**
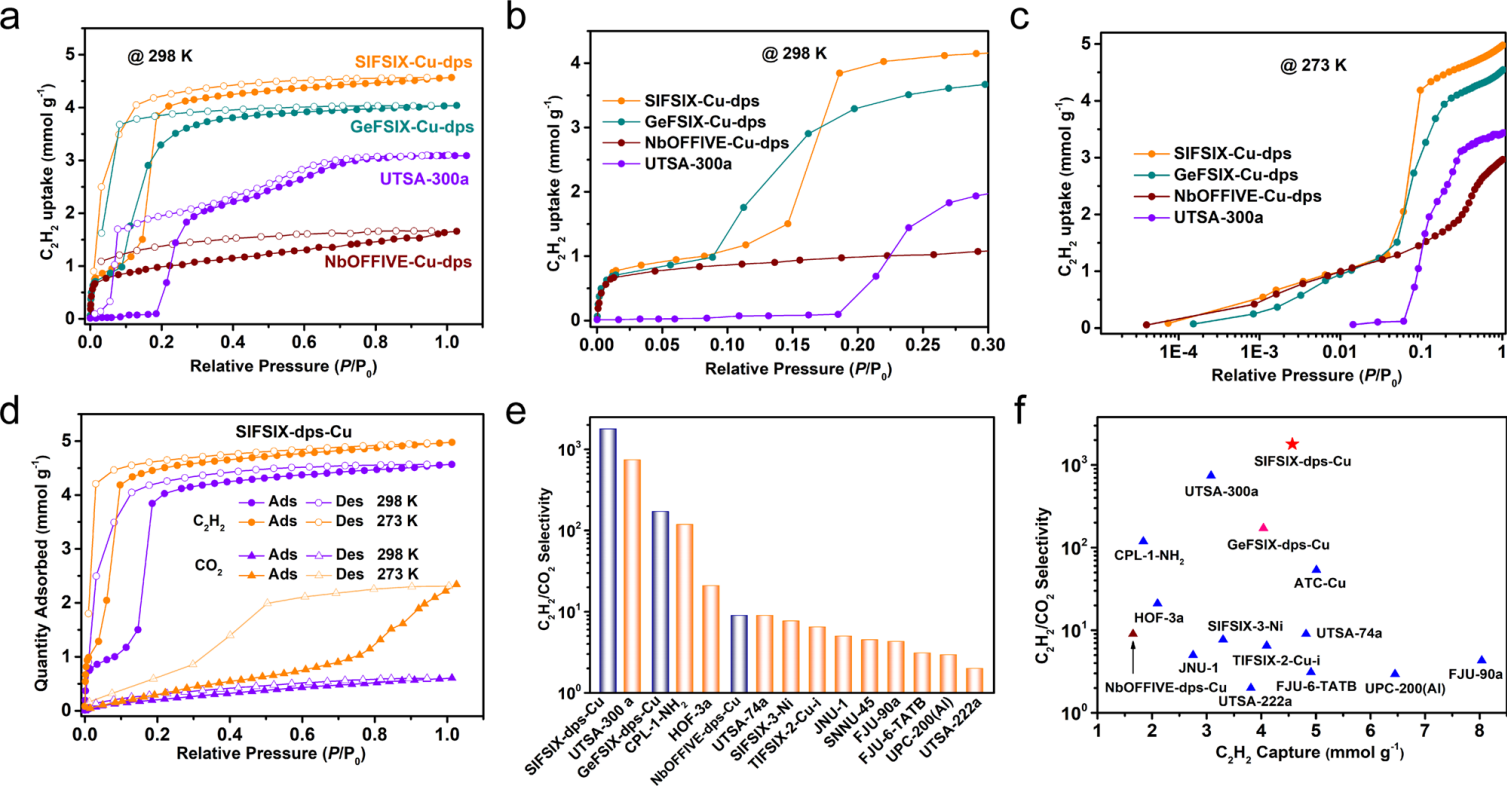
C_2_H_2_ and CO_2_ sorption in four isoreticular MOFs. The C_2_H_2_ sorption isotherms at **a** 0–1.0 bar and **b** 0–0.3 bar under 298 K. **c** The C_2_H_2_ sorption isotherms at logarithmic 0.01–1.0 bar of SIFSIX-dps-Cu, GeFSIX-dps-Cu, NbOFFIVE-dps-Cu, and UTSA-300a at 273 K. **d** the C_2_H_2_ and CO_2_ adsorption isotherms of SIFSIX-dps-Cu at 273 and 298 K. **e** comparison of IAST selectivity of representative MOFs for 50/50 C_2_H_2_/CO_2_. **f** Comparison about C_2_H_2_/CO_2_ selectivity and C_2_H_2_ capacity of representative MOFs at 298 K and 1 bar.

The permanent porosity of these dynamic layered MOFs is probed by CO_2_ adsorption isotherms at 195 K, and the Brunauer–Emmett–Teller specific surface area was determined as 358, 310, and 173 m^2^ g^−1^ for SIFSIX-dps-Cu, GeFSIX-dps-Cu, and NbOFFIVE-dps-Cu, respectively (Supplementary Figs. 9 and 10). It should be noted that NbOFFIVE-dps-Cu exhibits stepwise adsorption behavior with a smaller pore volume (0.09 cm^3^ g^−1^, at *P*/*P*_0_ ~0.25) before gate-opening as compared to those of SIFSIX-dps-Cu (0.20 cm^3^ g^−1^) and GeFSIX-dps-Cu (0.19 cm^3^ g^−1^), although it shows a potential total pore volume of 0.30 cm^3^ g^−1^. Thermogravimetric analysis revealed that these layered MOFs are stable up to 443 K (Supplementary Fig. 11). The XRD patterns showed that the activated structures can be restored to the as-synthesized state after being placed in the air for 24 h (Supplementary Figs. 2–4). Single-component equilibrium adsorption isotherms of C_2_H_2_ and CO_2_ were collected at 273, 298, and 323 K (Supplementary Figs. 12–14). The C_2_H_2_ uptake of SIFSIX-dps-Cu, GeFSIX-dps-Cu, and NbOFFIVE-dps-Cu was measured to be 4.57, 4.04, and 1.65 mmol g^−1^ at 298 K and 1.0 bar, respectively (Fig. 2a). The high C_2_H_2_ uptake of SIFSIX-dps-Cu (4.57 mmol g^−1^) outperforms many benchmark MOFs, including CPL-1-NH_2_ (1.84 mmol g^−1^)^41^, NTU-65 (3.36 mmol g^−1^)^34^, and JNU-1 (2.1 mmol g^−1^)^5^, see Supplementary Table 6. The sorption behaviors of these dynamic MOFs are similar to those in relevant literature^39,42^. The three MOF show similar C_2_H_2_ adsorption capacities (~0.85 mmol g^−1^) at the low-pressure region (Fig. 2b). This gate-opening phenomenon can be attributed by structural dynamics including the rotation of pyridinyl rings upon C_2_H_2_-loading. There are differences on the torsion angle between anion pillar and dps ligand before and after C_2_H_2_ loading in SIFSIX-dps-Cu (7°), GeFSIX-dps-Cu (6°), and NbOFFIVE-dps-Cu (1°), all of which are much smaller than that of UTSA-300a (17°) (Supplementary Figs. 15–16), matching well with corresponding static sorption results. The C_2_H_2_ threshold pressure for gate-opening of NbOFFIVE-dps-Cu is around 0.3 bar at 273 K, which was significantly higher than those of 0.06, 0.05, and 0.035 bar for UTSA-300a, GeFSIX-dps-Cu, and SIFSIX-dps-Cu, respectively (Fig. 2c). The dynamic adsorption behaviors were consistent with the trend of interlayer π–π stacking distances.

These results demonstrate that the subtle change of dynamic layered MOFs by substituting different pillared anions can greatly impact the C_2_H_2_ adsorption behavior, which would ultimately affect the selectivity of C_2_H_2_/CO_2_. At 298 K, NbOFFIVE-dps-Cu showed smooth CO_2_ adsorption of 1.10 mmol g^−1^. In contrast, GeFSIX-dps-Cu and SIFSIX-dps-Cu show minor CO_2_ uptake at low-pressure regions, although the latter shows stepwise CO_2_ uptake at 273 K and pressures above 0.7 bar (2.34 mmol g^−1^ at 1.0 bar, Fig. 2d). This might be ascribed to the gate-opening effect in the relatively flexible framework of SIFSIX-dps-Cu upon strong interactions with CO_2_. To investigate the adsorption phenomena for gas mixture, mixed-components adsorption isotherms of the three isoreticular MOFs for C_2_H_2_/CO_2_ (50/50, mol/mol) have also been collected (Supplementary Fig. 17). Compared with the single component sorption results, their adsorption capacity and threshold pressure are basically not changed, which confirms the preferential adsorption of C_2_H_2_ from C_2_H_2_/CO_2_ mixture^43^. Prompted by the dramatic uptake differences, the ideal adsorbed solution theory (IAST) was applied to qualitatively estimate the C_2_H_2_/CO_2_ selectivity, while the adsorption isotherms are fitted by the dual-site Langmuir–Freundlich equation with excellent accuracy (Supplementary Figs. 18–29 and Supplementary Table 7). As shown in Fig. 2e and Supplementary Fig. 30, the calculated equimolar C_2_H_2_/CO_2_ selectivity of NbOFFIVE-dps-Cu, GeFSIX-dps-Cu, and SIFSIX-dps-Cu at 298 K and 1.0 bar are 9, 172, and 1787, respectively. In particular, the selectivity of SIFSIX-dps-Cu is higher than that of UTSA-300a (743) and much higher than other benchmark MOFs (Supplementary Table 6), such as CPL-1-NH_2_ (119)^41^, ATC-Cu (53.6)^9^, TIFSIX-2-Cu-i (6.5)^44^, UTSA-74a (9)^45^, and UTSA-222a (2)^46^. Compared with those of adsorbents with high C_2_H_2_ adsorption capacities such as SNNU-45^47^, FJU-90a^48^, UPC-200(Al)^49^, and FJU-6-TATB^50^ (Fig. 2f), SIFSIX-dps-Cu and GeFSIX-dps-Cu are still out-performing.

### Modeling simulation studies

**Fig. 3:**
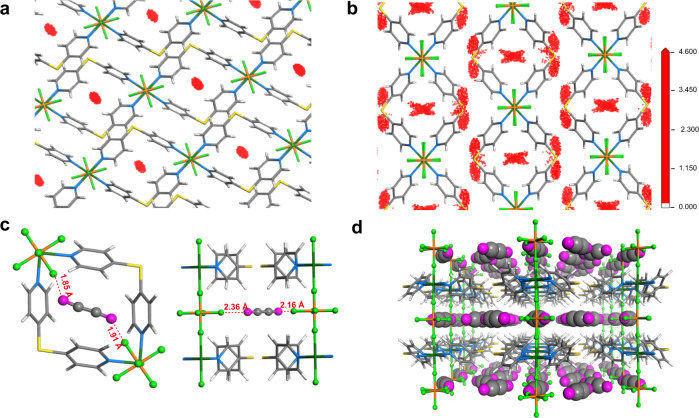
Simulated density distribution of C_2_H_2_ in SIFSIX-dps-Cu. C_2_H_2_ distributions in SIFSIX-dps-Cu by GCMC simulation **a** at 1 kPa and **b** at 100 kPa and 298 K, viewed along the (CuSiF_6_)_∞_ chains. **c** DFT-D calculated C_2_H_2_ binding mode in SIFSIX-dps-Cu. **d** Packing mode of C_2_H_2_-loaded SIFSIX-dps-Cu structure.

To investigate the potential C_2_H_2_ adsorption sites in these layered MOFs, dispersion-corrected density functional theory (DFT-D) and grand canonical Monte Carlo (GCMC) simulations are further carried out. Given that it is difficult to get the structures of intermediate states during the dynamic adsorption whereas the structural change during C_2_H_2_ loading is similar to UTSA-300, the activated or open frameworks were thus used for simulations. The distribution density of C_2_H_2_ was investigated firstly at 1 kPa, as shown in Fig. 3a, b and Supplementary Figs. 31–33, only C_2_H_2_ can be adsorbed in intralayer cavities (Site I) on all for isoreticular MOFs. As the loading pressure increased to 100 kPa, the interlayer cavities (Site II) became accessible by C_2_H_2_ stimuli (Fig. 3b). Such adsorption behavior is in line with that in UTSA-300 confirmed by neutron diffraction data^13^. In contrast, there is no gate-opening sorption for CO_2_ even at 100 kPa that may be attributed to the opposite molecular quadrupole moment (−13.4 × 10^−40^ C m^2^ for CO_2_ and +20.5 × 10^−40^ C m^2^ for C_2_H_2_)^13,51^. The C_2_H_2_ uptake of SIFSIX-dps-Cu, GeFSIX-dps-Cu, and NbOFIVE-dps-Cu was also evaluated by GCMC simulation showing the capacity of 4.40, 3.71, and 1.48 mmol g^−1^, respectively, which are comparable to their experimental uptakes. Moreover, DFT-D calculations provide insight into the adsorption behaviors and the calculation details are provided in the “Methods” section. The adsorption sites are similar in three dynamic layered MOFs, with C_2_H_2_ molecule bonded by four F atoms of two distinct fluorinated anion pillars at Site I and Site II via cooperative H∙∙∙F hydrogen-bond interactions (1.85‒2.36 Å, Fig. 3c and Supplementary Fig. 34). The static binding energy of SIFSIX-dps-Cu for C_2_H_2_ is calculated to be 60.3 and 67.5 kJ mol^−1^, respectively, which are also higher than those in GeFSIX-dps-Cu (58.5 kJ mol^−1^) and NbOFFIVE-dps-Cu (55.3 kJ mol^−1^). The abundant binding sites with high static binding energies are responsible for the outstanding C_2_H_2_ uptake of SIFSIX-dps-Cu (Fig. 3d). In contrast, CO_2_ in SIFSIX-dps-Cu interacts with pore surface through weak intermolecular interactions like electrostatic interactions (F^δ−^∙∙∙C^δ+^ 2.91‒3.63 Å, Supplementary Fig. 35). The change in torsion angle between anion pillar and dps ligand caused by CO_2_ loading is about 18^o^, which is larger than that for C_2_H_2_ loading (7^o^), in line with higher gate-opening pressure for CO_2_ sorption. The experimental isosteric adsorption enthalpy (*Q*_st_) at zero-coverage for C_2_H_2_ in SIFSIX-dps-Cu is 60.5 kJ mol^−1^ (Supplementary Fig. 36), slightly higher than the *Q*_*st*_ of GeFSIX-dps-Cu (56.3 kJ mol^−1^) and NbOFFIVE-dps-Cu (53.6 kJ mol^−1^).

### Transient breakthrough experiments

**Fig. 4:**
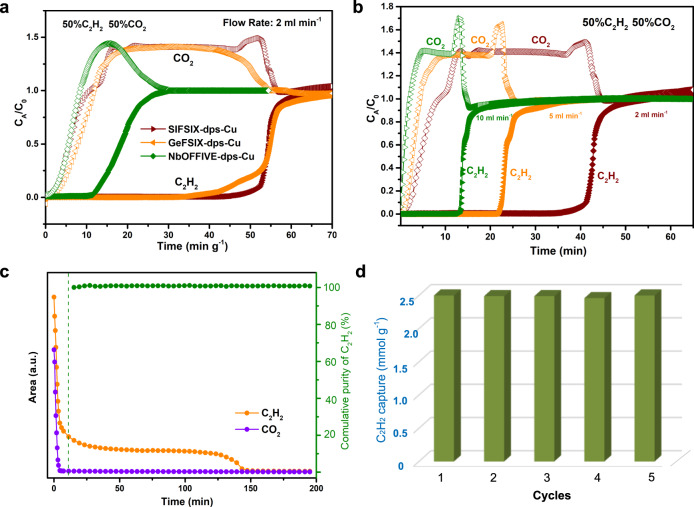
C_2_H_2_/CO_2_ separation. Breakthrough curves of C_2_H_2_/CO_2_ (50/50) in **a** representative MOFs with a 2 ml min^−1^ flow rate at 298 K. **b** The breakthrough curves with different flow rates at 298 K. **c** The signals of desorbed gases from SIFSIX-dps-Cu. **d** Cycling stability of SIFSIX-dps-Cu for C_2_H_2_/CO_2_ (50/50) separation.

To confirm the practical C_2_H_2_/CO_2_ separation performances of these isoreticular MOFs, experimental breakthrough experiments were conducted with SIFSIX-dps-Cu, GeFSIX-dps-Cu, and NbOFFIVE-dps-Cu at 298 K. Binary mixture (C_2_H_2_/CO_2_, 50/50, v/v) were injected into a packed column with a flow rate of 2.0 ml min^−1^ and the clean separations of C_2_H_2_/CO_2_ mixtures were realized by all dynamic layered materials (Supplementary Figs. 37 and 38). As expected, SIFSIX-dps-Cu exhibits the best C_2_H_2_/CO_2_ separation performance. In Fig. 4a, CO_2_ broke through the bed quickly after feeding the gas mixture into the fixed adsorption column, whereas the C_2_H_2_ was retained in the adsorption bed for 53 min g^−1^. This value is comparable to GeFSIX-dps-Cu (50 min g^−1^) and significantly outperforms NbOFFIVE-dps-Cu (14 min g^−1^) and UTSA-300a (12 min g^−1^) at similar conditions. For small roll-up of the breakthrough curves for CO_2_ in SIFSIX-dps-Cu, it can be attributed to the desorption of CO_2_ induced by C_2_H_2_, which indicates a minor co-adsorption of CO_2_ during the dynamic capture process and finally displaced by C_2_H_2_. That phenomenon is consistent with the minor CO_2_ uptake from single-component sorption isotherms. The C_2_H_2_ and CO_2_ adsorption kinetics in SIFSIX-dps-Cu showed no significant CO_2_ uptake under a sufficiently long period of time but rapid C_2_H_2_ saturation with a capacity of 3.72 mmol g^−1^ at 0.5 bar and 298 K, indicating that CO_2_ cannot diffuse into the framework of SIFSIX-dps-Cu (Supplementary Fig. 39). At 5 and 10 ml min^−1^, the roll-up phenomenon becomes significant that might induce by faster gas displacement at a higher flow rate (Fig. 4b). Moreover, the adsorbent could be completely regenerated with a He flows rate of 10 ml min^−1^ at 298 K. In the desorption process, CO_2_ will be eluted immediately, and then high-purity C_2_H_2_ product (≥99.9%) can be collected in the interior of the packed column for 137 min (Fig. 4c). This result also demonstrated the negligible co-adsorption of CO_2_. For the minor inconsistency between the CO_2_ breakthrough curve and equilibrium CO_2_ isotherm of NbOFFIVE-dps-Cu, it can be attributed to the preferential capture of C_2_H_2_ from the dynamic gas flow by this MOF that inhibits CO_2_ adsorption, as indicated by distinctly different adsorption heats (C_2_H_2_: 53.6 kJ mol^−1^, CO_2_: 28.8 kJ mol^−1^) and dual-components adsorption result (Supplementary Fig. 17). The corresponding desorption curves of NbOFFIVE-dps-Cu in the regeneration process after C_2_H_2_/CO_2_ breakthrough have also confirmed negligible CO_2_ co-adsorption (Supplementary Fig. 40).

Furthermore, the C_2_H_2_ productivity of SIFSIX-dps-Cu calculated from the breakthrough curve is 2.48 mmol g^−1^, which outperforms those of CPL-1-NH_2_ (1.38 mmol g^−1^)^41^, and is comparable to SIFSIX-3-Ni (2.5 mmol g^−1^)^44^. And the C_2_H_2_ productivity of GeFSIX-dps-Cu and NbOFFIVE-dps-Cu was calculated to be 2.36 and 0.83 mmol g^−1^, respectively, which are significantly superior to that of UTSA-300a (0.77 mmol g^−1^)^13^. In particular, these results validate the flexible-robust pore space of these dynamic MOFs for simultaneous high C_2_H_2_ capacity and C_2_H_2_/CO_2_ selectivity. It should be noted that the C_2_H_2_ productivity of the three MOFs calculated from breakthrough curves is lower than those of corresponding maximum adsorption capacity (4.57, 4.04, and 1.65 mmol g^−1^ for SIFSIX-dps-Cu, GeFSIX-dps-Cu, and NbOFFIVE-dps-Cu, respectively), which might be attributed to the inter-framework diffusional resistances. For practical industrial applications, the adsorbents are expected to show good recyclability. Thus, five successive C_2_H_2_/CO_2_ dynamic breakthrough experiments were carried out on SIFSIX-dps-Cu, GeFSIX-dps-Cu, and NbOFFIVE-dps-Cu at three flow rates, and negligible deteriorations in breakthrough time and working capacity during five cycles indicates their outstanding recyclability (Fig. 4d and Supplementary Fig. 38). Furthermore, PXRD and sorption studies upon various conditions show that SIFSIX-dps-Cu can maintain its crystallinity in water, several organic solvents or under moisture for at least 7 days (Supplementary Fig. 41).

## Discussion

The adsorption behaviors in a series of isoreticular MOFs have been successfully controlled as a result of pore size adjustment through altering the anionic linkers, and thus realizing highly efficient C_2_H_2_/CO_2_ separations. After tuning with dual functionality namely appropriate pore size and specific functional sites, novel adsorbent variants can exhibit both excellent C_2_H_2_ uptake and C_2_H_2_/CO_2_ selectivity at ambient conditions. The precise pore engineering would apply to many other MOFs regarding basic principles in MOF chemistry. This work illustrates a good example to realize high-performance materials for molecular recognition and will inspire future designs on novel porous materials.

## Methods

All reagents were purchased from commercial companies and used without further purification.

### Synthesis of SIFSIX-dps-Cu

The sample was prepared according to ref. ^39^, and reproduced here for completeness. A methanol solution (5.0 ml) of dps (0.054 g, 0.286 mmol) was slowly added to an aqueous solution (5.0 ml) of Cu (BF_4_)_2_·*x*H_2_O (0.066 g, 0.26 mmol) and (NH_4_)_2_SiF_6_ (0.046 g, 0.26 mmol) at room temperature, the mixture was kept undisturbed at room temperature for 48 h. Then the purple powder was washed with methanol and dried under a high vacuum at room temperature for 24 h.

### Synthesis of GeFSIX-dps-Cu

A methanol solution (5.0 ml) of dps (0.054 g, 0.286 mmol) was slowly added to an aqueous solution (5.0 ml) of Cu (BF_4_)_2_·*x*H_2_O (0.066 g, 0.26 mmol) and (NH_4_)_2_GeF_6_ (0.058 g, 0.26 mmol) at room temperature, the mixture was kept undisturbed at room temperature for 48 h. Then the purple powder was washed with methanol and dried under a high vacuum at room temperature for 24 h.

The single crystals of GeFSIX-dps-Cu were synthesized by the slow diffusion of a methanol solution (1.0 ml) of dps (0.011 g, 0.057 mmol) into an aqueous solution (1.0 ml) of Cu(BF_4_)_2_·*x*H_2_O (0.013 g, 0.052 mmol) and (NH_4_)_2_GeF_6_ (0.012 g, 0.052 mmol) at room temperature in a watch glass without stirring. Specifically, 0.5 ml of 1:1 methanol/H_2_O was layered between the top and bottom solutions to slow the rate of reaction. Light purple and rectangular prismatic crystals formed after 4 days.

### Synthesis of NbOFFIVE-dps-Cu

A methanol solution (5.0 ml) of dps (0.054 g, 0.286 mmol) was slowly added to an aqueous solution (5.0 ml) of Cu (BF_4_)_2_·*x*H_2_O (0.066 g, 0.26 mmol) and (NH_4_)_2_NbF_6_ (0.059 g, 0.26 mmol) at room temperature, the mixture was kept undisturbed at room temperature for 48 h. Then the purple powder was washed with methanol and dried under a high vacuum at room temperature for 24 h.

The single crystals of NbOFFIVE-dps-Cu were synthesized by the slow diffusion of a methanol solution (1.0 ml) of dps (0.011 g, 0.057 mmol) into an aqueous solution (1.0 ml) of Cu(BF_4_)_2_·*x*H_2_O (0.013 g, 0.052 mmol) and (NH_4_)_2_NbF_6_ (0.012 g, 0.052 mmol) at room temperature in a watch glass without stirring. Specifically, 0.5 ml of 1:1 methanol/H_2_O was layered between the top and bottom solutions to slow the rate of reaction. Light purple and rectangular prismatic crystals formed after 4 days.

### X-ray diffraction structure analysis

PXRD patterns were measured by a PANalytical Empyrean Series 2 diffractometer with Cu Kα radiation with a step size of 0.0167°, a scan time of 15 s per step, and 2*θ* ranging from 5 to 90° at room temperature.

### Single-crystal X-ray diffraction

Single crystal X-ray diffraction data for GeFSIX-dps-Cu and NbOFFIVE-dps-Cu were collected at 193(2) K on a Bruker-AXS D8 VENTURE diffractometer equipped with a PHOTON-100/CMOS detector (GaKα, *λ* = 1.3414 Å). Indexing was performed using APEX2. SaintPlus 6.01 was used to complete data integration and reduction. The multi-scan method implemented in SADABS was used to conduct absorption correction. XPREP implemented in APEX2.1 was used to determine the space group. The structures were solved by direct methods and refined by nonlinear least-squares on *F*^2^ (method) with SHELXL-97 contained in APEX2, OLEX2 v1.1.5, and WinGX v1.70.01 program packages. All non-hydrogen atoms were refined anisotropically. The Squeeze routine implemented in Platon was used to treat the contribution of disordered solvent molecules as diffuse.

### The thermogravimetric analysis (TGA)

The thermogravimetric analysis (TGA) data were collected in a NETZSCH Thermogravimetric Analyzer (STA2500) from 25 to 700 °C with a heating rate of 10 °C/min.

### Gas sorption measurements

A Micromeritics ASAP 2460 adsorption apparatus was used to measure gas adsorption isotherms. In order to remove all the guest solvents in the framework, the fresh powder samples were evacuated under a high vacuum at room temperature (298 K) for 72 h. Liquid nitrogen and dry ice-acetone bath were used for adsorption isotherms at 77 or 196 K. The helium gas was used to determine the free space of the system. The degas procedure was repeated on the same sample between measurements for 24 h.

### Calculation of isosteric heat of adsorption (*Q*_st_)

The experimental adsorption enthalpy (*Q*_st_) was applied to evaluate the binding strength between adsorbent and adsorbate, defined as

1 $${Q}_{{{{{\rm{st}}}}}}={{{{{\rm{RT}}}}}}^{2}\left(\frac{\partial {lnp}}{\partial T}\right)$$

The isosteric heat of adsorption, *Q*_st_ is determined using the pure component isotherm fits using the Clausius–Clapeyron equation, where *Q*_st_ (kJ mol^−1^) is the isosteric heat of adsorption, *T* (K) is the temperature, *p* (kPa) is the pressure, and *R* is the gas constant.

### DFT calculations

The first-principles DFT calculations were proceeded using the Quantum-Espresso package^52^. van der Waals interactions were illustrated by the calculation with a semiempirical addition of dispersive forces to the conventional DFT^53^. Vanderbilt-type ultrasoft pseudopotentials and generalized gradient approximation (GGA) with a Perdew–Burke–Ernzerhof were used for exchange-correlation. We found that cutoff energy of 544 eV and a 2 × 2 × 2 k-point mesh (generated using the Monkhosrt–Pack scheme) were enough for the total energy to converge within 0.01 meV/atom. Fully open structures were used for the calculations of binding energy. First, the structure was optimized. Afterward, the various guest gas molecules were placed to various locations of the pore structure, followed by a full structural relaxation. To gain the gas binding energy, an isolated gas molecule that was placed in a supercell (with the same cell dimensions as the MOF crystal) was also relaxed. The static binding energy (at *T* = 0 K) was then calculated using *E*_B_ = *E*(MOF) + *E*(gas) − *E*(MOF + gas).

### GCMC simulations

The GCMC simulations which were carried out to investigate the adsorbed capacity of wavy layered MOFs for C_2_H_2_/CO_2_ at 298 K from 0.001 to 100 kPa were performed by sorption code in MS software. Activated structures were used for the simulation of adsorption before gate-opening, whereas fully open structures were used for the simulation of adsorption after gate-opening. We used a simulation box with a 1 × 1 × 1 crystallographic unit cell. During the simulations, in order to guarantee the equilibration and to sample the desired properties, 4 × 10^6^ steps were performed. In all simulations, a rigid framework assumption was employed. We describe the interactions using the Dreiding forcefield parameter12, Lenard–Jones 12-6 potential was used to depict the van der Waals interaction with a cutoff of 15.5 Å^12^.

The GCMC simulations were performed in the *NVT* ensemble to calculate the isosteric heats of adsorption *Q*_st_. The internal energy Δ*U* was computed during the simulation, which is directly related to *Q*_st_. The isosteric heat of adsorption *Q*_st_ was calculated from

2 $${Q}_{{{{{\rm{st}}}}}}={{{{{\rm{RT}}}}}}-\frac{\left\langle {U}_{{ff}}N\right\rangle -\left\langle {U}_{{ff}}\right\rangle \left\langle N\right\rangle }{\left\langle {N}^{2}\right\rangle -\left\langle N\right\rangle \left\langle N\right\rangle }-\frac{\left\langle {U}_{{sf}}N\right\rangle -\left\langle {U}_{{sf}}\right\rangle \left\langle N\right\rangle }{\left\langle {N}^{2}\right\rangle -\left\langle N\right\rangle \left\langle N\right\rangle }$$

where *R* is the gas constant, *N* is the number of molecules adsorbed, and 〈 〉 indicates the ensemble average. The *U*_ff_ in the first and second terms are the contributions from the molecular thermal energy and adsorbate-adsorbate interaction energy, respectively. The *U*_sf_ in the third term is the contribution from the adsorbent–adsorbate interaction energy.

### IAST calculations

In order to calculate the selective sorption performance for SIFSIX-dps-Cu, GeFSIX-dps-Cu, and NbOFFIVE-dps-Cu toward the separation of binary mixed gases, the fitting of single-component C_2_H_2_ and CO_2_ adsorption isotherms were carried out based on the DSLF model. The fitting parameters of the DSLF equation are displayed in Supplementary Table 7. Adsorption isotherms and gas selectivities of mixed C_2_H_2_/CO_2_ (50/50, v/v) at 298 K were predicted using the IAST. The results are shown in Supplementary Figs. 22–27.

DSLF model is listed below

3 $$N={N}_{1}^{{{\max }}}\times \frac{{b}_{1}{p}^{1/n1}}{1+{b}_{1}{p}^{1/n1}}+{N}_{2}^{{{\max }}}\times \frac{{b}_{2}{p}^{1/{{{{{\rm{n2}}}}}}}}{1+{b}_{2}{p}^{1/{{{{{\rm{n2}}}}}}}}$$

Where *p* (unit: kPa) is the pressure of the bulk gas at equilibrium with the adsorbed phase, *N* (unit: mol/kg) is the adsorbed amount per mass of adsorbent, *N*_1_^max^ and *N*_2_^max^ (unit: mmol/g) are the saturation capacities of two different sites, *b*_1_ and *b*_2_ (unit: 1/kPa) are the affinity coefficients of these sites, and *n*_1_ and *n*_2_ represent the deviations from an ideal homogeneous surface.

The adsorption selectivity for the mixtures C_2_H_2_/CO_2_ is defined by

4 $${S}_{{{{{\rm{ads}}}}}}=\frac{{q}_{1}/{q}_{2}}{{p}_{1}/{p}_{2}}$$

were calculated according to the IAST model proposed by Myers^54,55^, in the above equation, *q*_1_ and *q*_2_ are the absolute component loadings of the adsorbed phase in the mixture. These component loadings are also termed uptake capacities.

### Transient breakthrough experiments

The breakthrough experiments were carried out in a homemade apparatus. The feeding streams are gas-mixtures of 50/50 (v/v) C_2_H_2_/CO_2_ with a flow rate of 2 ml min^−1^ (298 K and 1.01 bar). The mass packed in the sample holder was: SIFSIX-dps-Cu (0.7881 g), GeFSIX-dps-Cu (0.7624 g), and NbOFFIVE-dps-Cu (0.7313 g). Activated samples were packed into a Φ6.3 × 140 mm stainless steel column. A carrier gas (He ≥ 99.999%) was used to purge the adsorption bed for about 12 h at room temperature. A mass flow meter was used to regulate the gas flows, and the outlet gas from the column was monitored using mass spectrometry (Hidden, UK).) After each separation test, the sample was regenerated with a He flow of 15 ml min^−1^ at 298 K. Ultrahigh purity grade helium (99.999%), acetylene (>99%), carbon dioxide (99%), and nitrogen (99.999%) were purchased from Nanchang Guoteng Gas Co., Ltd. (China).

## Supplementary information

Supplementary Information

Peer Review File

## Data Availability

All data supporting the finding of this study are available within this article and its Supplementary Information. Crystallographic data for the structures in this article have been deposited at the Cambridge Crystallographic Data Centre under deposition Nos. CCDC 2060207 (GeFSIX-dps-Cu) and 2060208 (NbOFFIVE-dps-Cu). Copies of the data can be obtained free of charge from www.ccdc.cam.ac.uk/data_request/cif. Source data that support the findings of this study are available from the corresponding author upon request. Additional graphics, model fitting, and calculations are available within its Supplementary Information.
