# Supplementary material for: Fine pore engineering in a series of isoreticular metal-organic frameworks for efficient C2H2/CO2 separation
Source: Nat Commun. 2022 Jan 11;13:200. doi: 10.1038/s41467-021-27929-7 (PMC8752597; doi:10.1038/s41467-021-27929-7)
Supplement: Supplementary file 1 — Supplementary Information [file 41467_2021_27929_MOESM1_ESM.pdf]

*Supplementary Information*

# Fine Pore Engineering in a Series of Isoreticular Metal-Organic Frameworks for Efficient $C_2H_2/CO_2$ Separation

Jun Wang[1], Yan Zhang[2], Yun Su[1], Xing Liu[1], Peixin Zhang[3], Rui-Biao Lin[4*], Shixia Chen[1], Qiang Deng[1], Zheling Zeng[1], Shuguang Deng[5*], and Banglin Chen[6*]

[1] School of Resource, Environmental and Chemical Engineering, Nanchang University, Nanchang, 330031, Jiangxi, PR China

[2] Jiangxi University of Chinese Medicine, Nanchang, 330031, Jiangxi, PR China

[3] Key Laboratory of Biomass Chemical Engineering of Ministry of Education, College of Chemical and Biological Engineering, Zhejiang University, Hangzhou, 310027, Zhejiang, PR China

[4] MOE Key Laboratory of Bioinorganic and Synthetic Chemistry, School of Chemistry, Sun Yat-Sen University, Guangzhou, 510006, Guangdong, China

[5] School for Engineering of Matter, Transport and Energy, Arizona State University, 551 E. Tyler Mall, Tempe, AZ 85287, USA

[6] Department of Chemistry, University of Texas at San Antonio One UTSA Circle, San Antonio, TX 78249-0698, USA

*Corresponding author:

Porf. B. Chen, E-mail: banglin.chen@utsa.edu

Porf. S. Deng, E-mail: shuguang.deng@asu.edu

Porf. R.-B. Lin, E-mail: linruibiao@mail.sysu.edu.cn

## General Information and Procedures

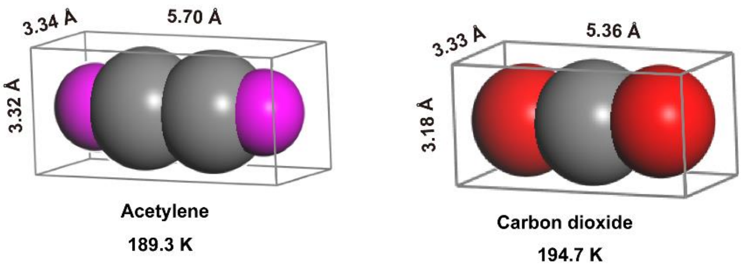


**Supplementary Figure 1.** The structures and size of $C_2H_2$ and $CO_2$.

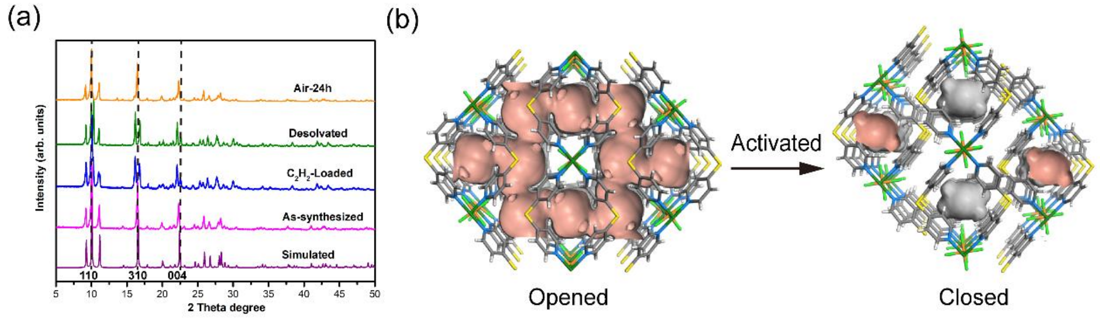


**Supplementary Figure 2.** (a) The powder X-ray diffraction patterns of SIFSIX-dps-Cu, and (b) structural transformation between as-synthesized phase to desolvated one. For SIFSIX-dps-Cu, after activation, the original peaks at 10°, 16.6°, and 22.5° showed significant changes that might be assigned to the sliding and shrinking of 2D coordination layers. The space group changes from *Ibam* to *P*2/*n* during the phase transformation. a dense structure with dispersed 0D cavities was thus obtained. Upon $C_2H_2$-loading, PXRD patterns and structure configurations reverse to the corresponding open state.

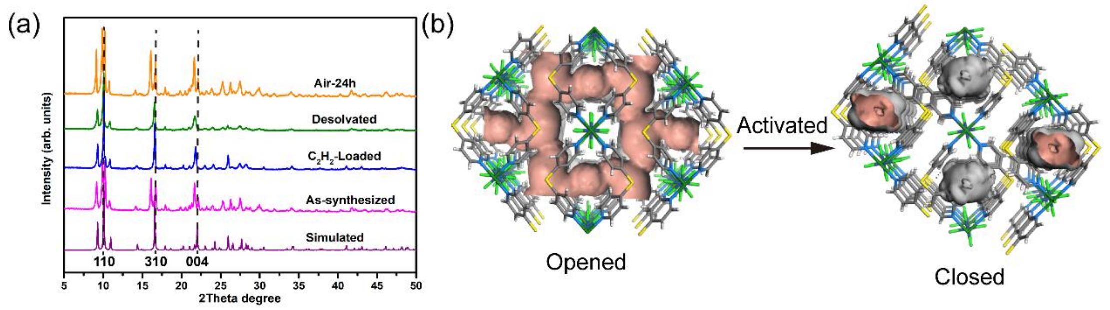


**Supplementary Figure 3.** (a) The powder X-ray diffraction patterns of GeFSIX-dps-Cu, and (b) structural transformation between as-synthesized phase to desolvated one. The changes of PXRD patterns (significantly at 10°, 16°, and 21.5°) and corresponding structural transformation in GeFSIX-dps-Cu are similar to those of SIFSIX-dps-Cu.

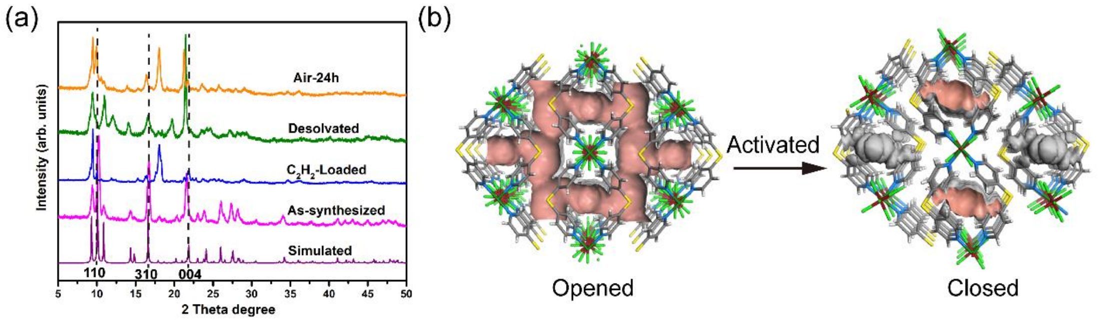


**Supplementary Figure 4.** (a) The powder X-ray diffraction patterns of NbOFFIVE-dps-Cu, and (b) structural transformation between as-synthesized phase to desolvated one. The changes of PXRD patterns (significantly at 10°, 16.5°, and 18°) and corresponding structural transformation in NbOFFIVE-dps-Cu are similar to those of SIFSIX-dps-Cu.

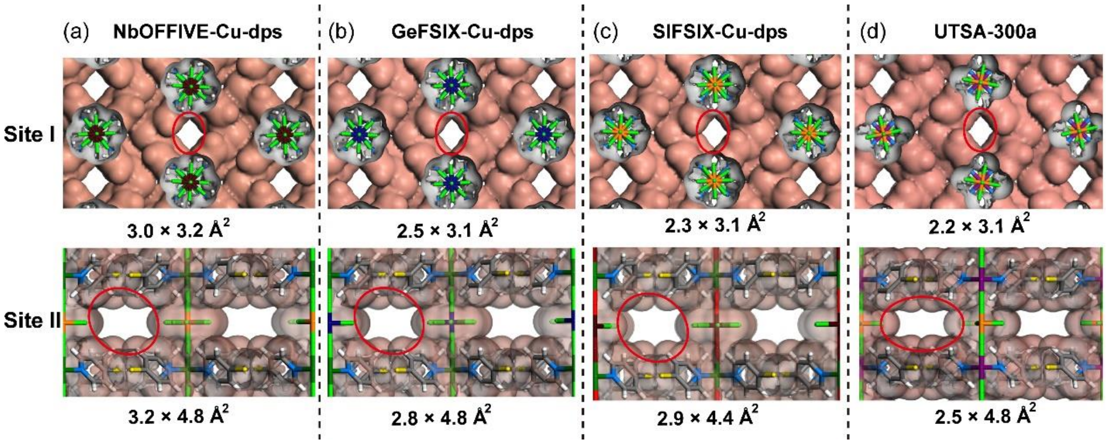


**Supplementary Figure 5.** Schematic representations of the layered structure; the intralayer and interlayer structures of (a) NbOFFIVE-dps-Cu, (b) GeFSIX-dps-Cu, (c) SIFSIX-dps-Cu, and (d) UTSA-300. Color code: Cu, green; F, light green; S, bright yellow; N, light blue; C, gray; Si, orange; Ge, blue; Nb, navy blue; and solvent molecules are omitted for clarity.

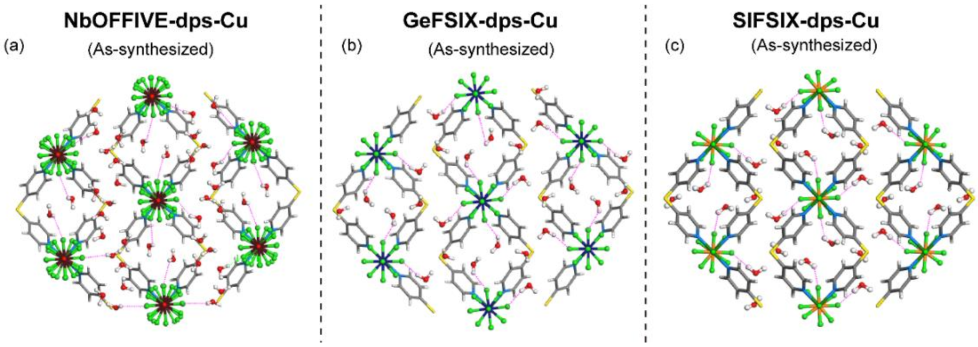


**Supplementary Figure 6.** The as-synthesized structures of (a) NbOFFIVE-dps-Cu, (b) GeFSIX-dps-Cu, and (c) SIFSIX-dps-Cu.

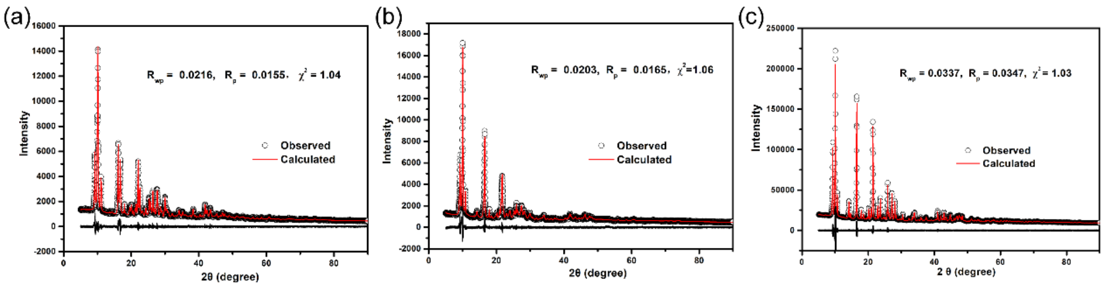


**Supplementary Figure 7.** Powder X-ray diffraction patterns for Rietveld refinement of activated (a) SIFSIX-dps-Cu, (b) GeFSIX-dps-Cu, and (c) NbOFFIVE-dps-Cu. Rietveld structural refinement was performed on the PXRD data using the GSAS package. Due to a large number of atoms in the crystal unit cell, the ligand molecule and the gas molecule were both treated as rigid bodies during the Rietveld refinement, with the molecule orientation and center of mass freely refined. Final refinement on the positions/orientations of the rigid bodies, thermal factors, occupancies, lattice parameters, background, and profiles converge with satisfactory R factors.

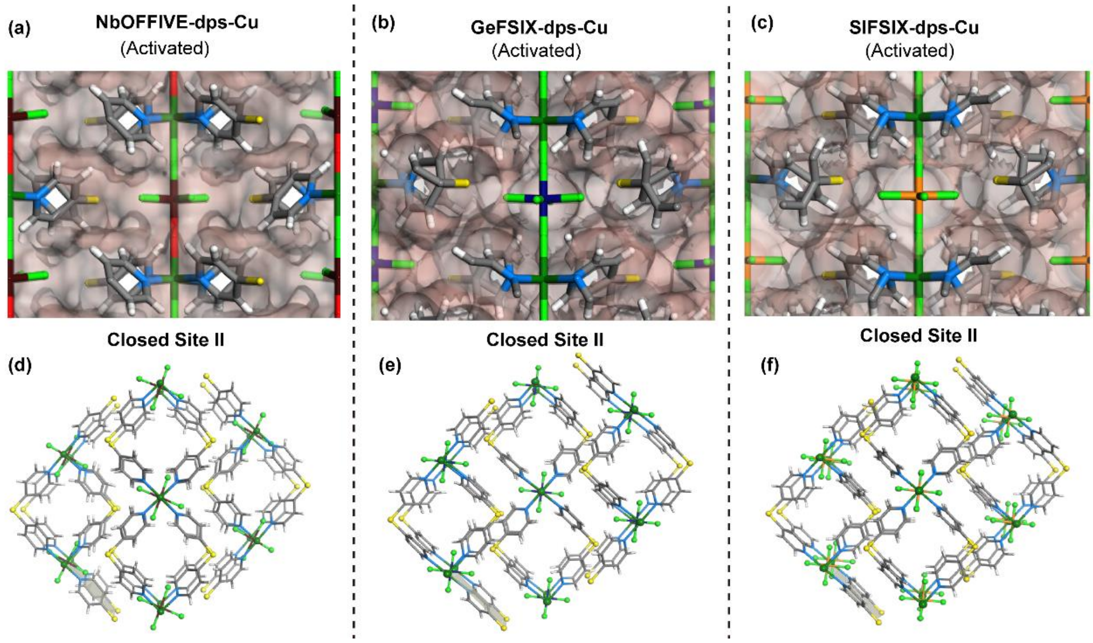


**Supplementary Figure 8.** The activated cavities of (a,d) NbOFFIVE-dps-Cu, (b,e) GeFSIX-dps-Cu, and (c,f) SIFSIX-dps-Cu.

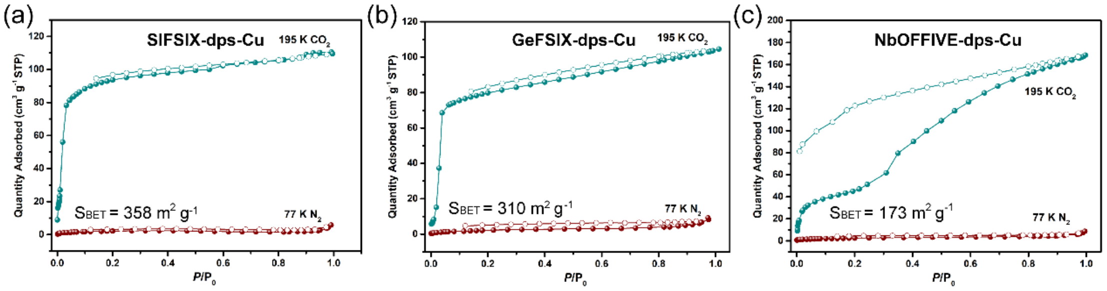


**Supplementary Figure 9.** Low-temperature $N_2$ and $CO_2$ sorption isotherms of (a) SIFSIX-dps-Cu, (b) GeFSIX-dps-Cu, and (c) NbOFFIVE-dps-Cu.

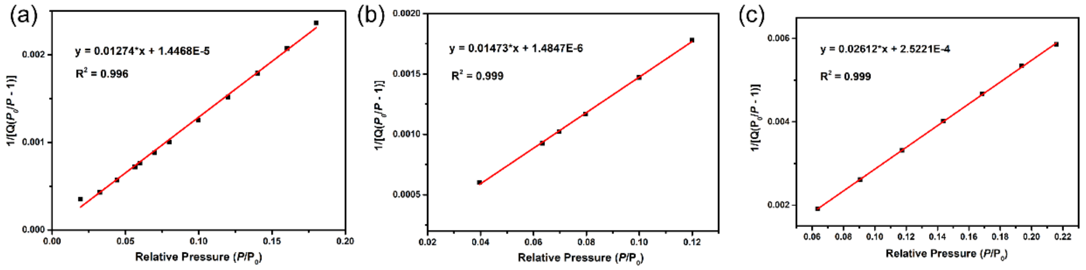


**Supplementary Figure 10**. Plots for calculation of BET surface area, based on $CO_2$ adsorption isotherms of (a) SIFSIX-dps-Cu, (b) GeFSIX-dps-Cu, and (c) NbOFFIVE-dps-Cu at 195 K.

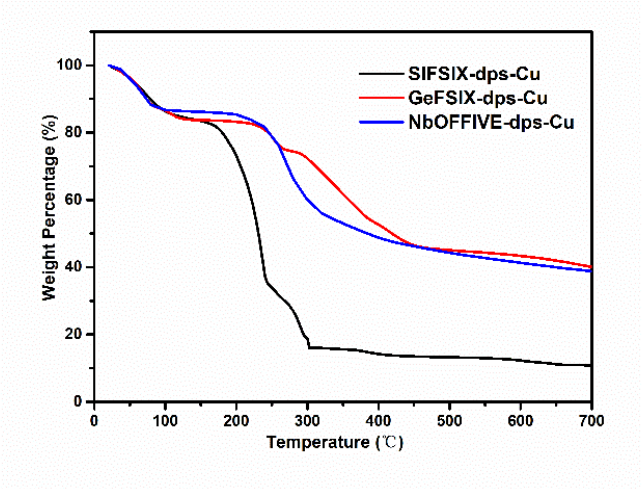


**Supplementary Figure 11**. TGA curve of SIFSIX-dps-Cu, GeFSIX-dps-Cu, and NbOFFIVE-dps-Cu.

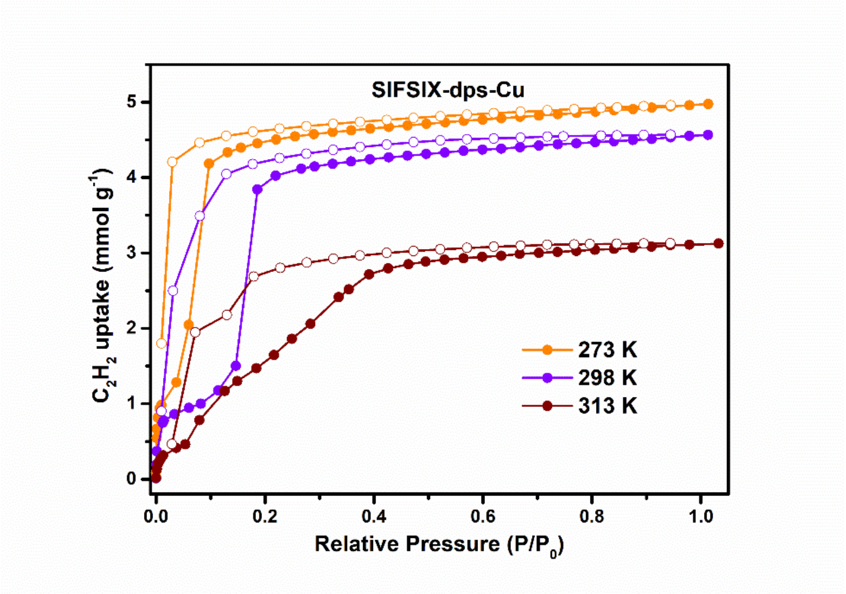


**Supplementary Figure 12.** $C_2H_2$ isotherms of SIFSIX-dps-Cu at 273 K, 298 K, and 313 K.

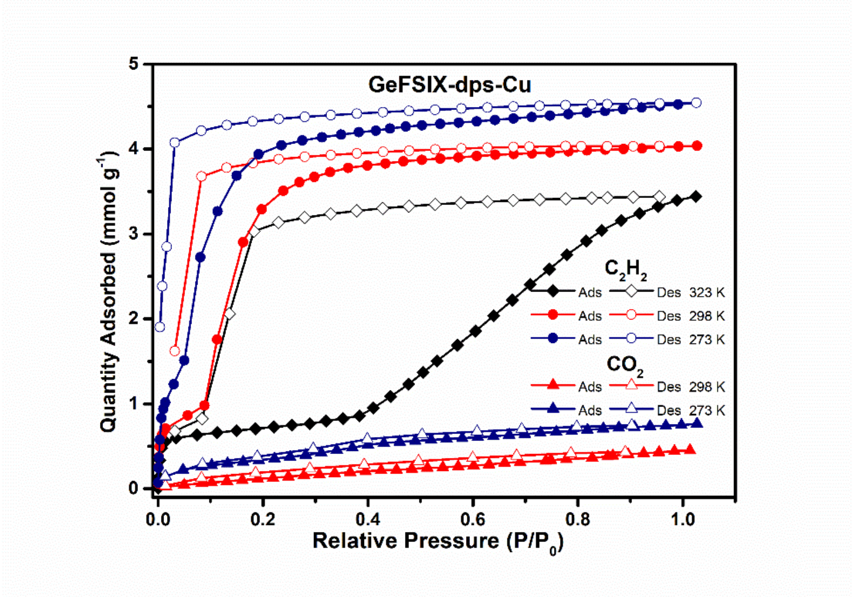


**Supplementary Figure 13.** $C_2H_2$ and $CO_2$ isotherms of GeFSIX-dps-Cu at 273 K, 298 K, and 323 K.

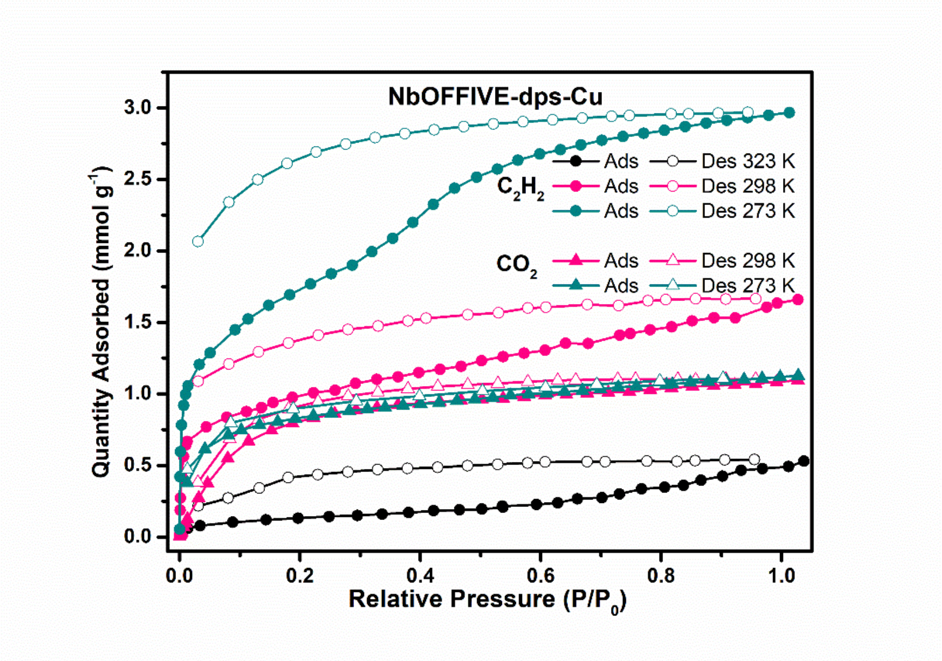


**Supplementary Figure 14.** $C_2H_2$ and $CO_2$ isotherms of NbOFFIVE-dps-Cu at 273 K, 298 K, and 323 K.

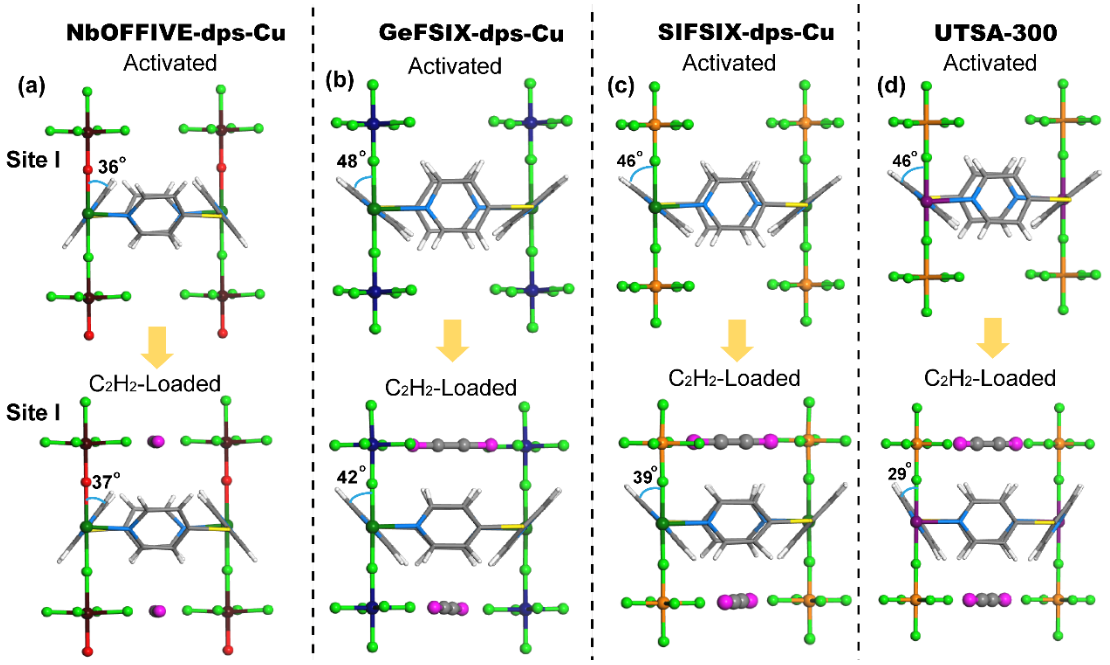


**Supplementary Figure 15.** The active and $C_2H_2$-loaded structures of (a) NbOFFIVE-dps-Cu, (b) GeFSIX-dps-Cu, (c) SIFSIX-dps-Cu, and (d) UTSA-300. The torsion angle was measured from C–N bond on pyridinyl ring to Cu–F/O bond.

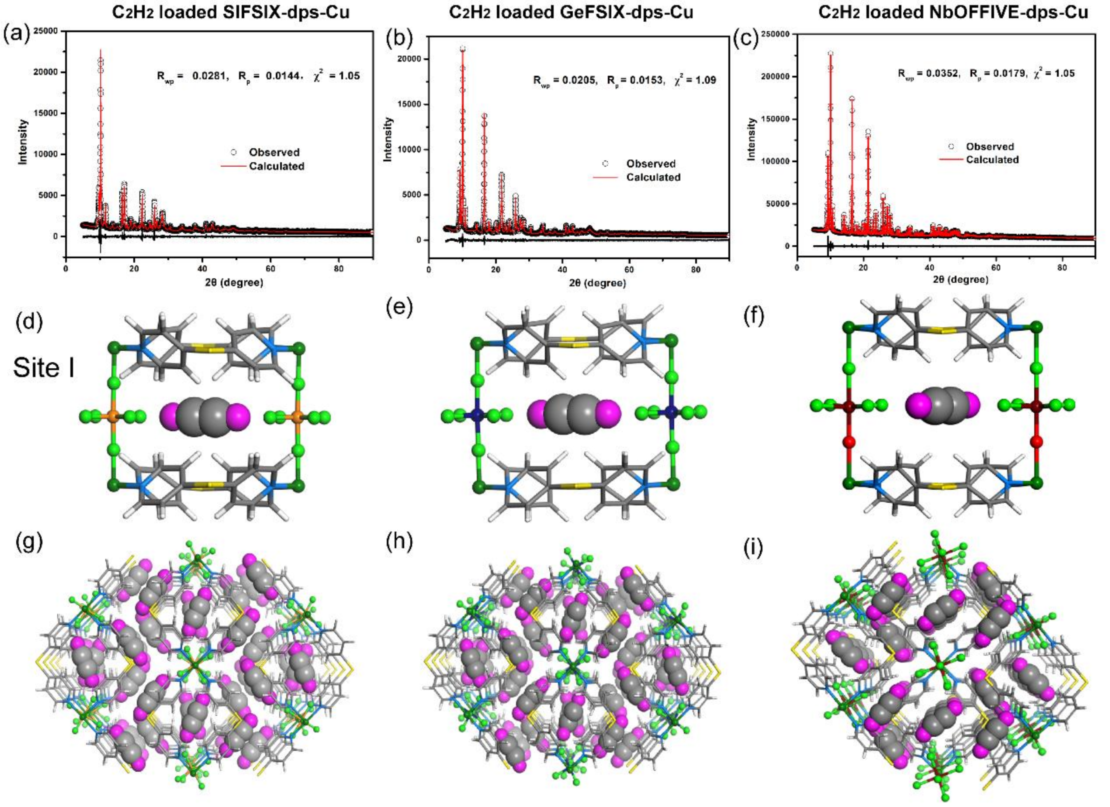


**Supplementary Figure 16.** Powder X-ray diffraction patterns for Rietveld refinement of $C_2H_2$-loaded (a) SIFSIX-dps-Cu, (b) GeFSIX-dps-Cu, and (c) NbOFFIVE-dps-Cu. The $C_2H_2$ accumulation patterns at Site I and Site II of $C_2H_2$-loaded (d,g) SIFSIX-dps-Cu, (e,h) GeFSIX-dps-Cu, and (f,i) NbOFFIVE-dps-Cu.

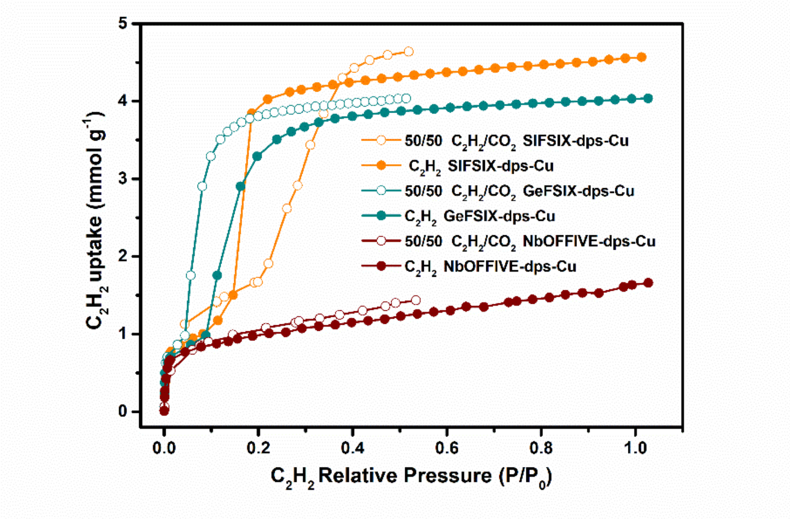


**Supplementary Figure 17**. Sorption isotherms of SIFSIX-dps-Cu, GeFSIX-dps-Cu, and NbOFFIVE-dps-Cu for pure $C_2H_2$ (solid symbol) and equimolar mixture of $C_2H_2/CO_2$ (50/50, mol/mol, empty symbol) at 298 K, respectively.

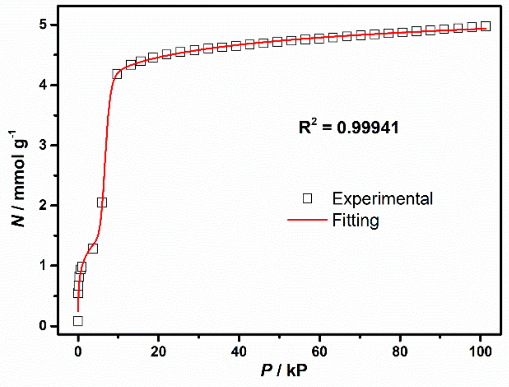


**Supplementary Figure 18.** DSLF fitting of the $C_2H_2$ sorption data at 273 K and 1 bar on SIFSIX-dps-Cu.

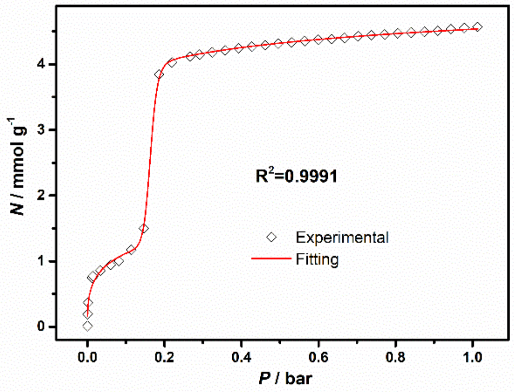


**Supplementary Figure 19.** DSLF fitting of the $C_2H_2$ sorption data at 298 K and 1 bar on SIFSIX-dps-Cu.

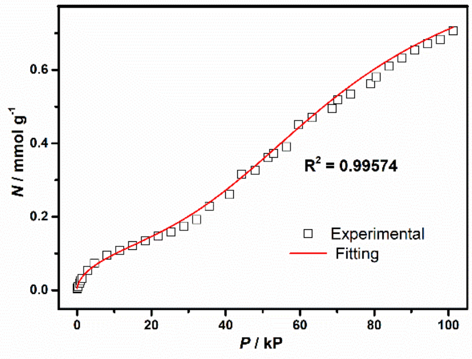


**Supplementary Figure 20.** DSLF fitting of the $C_2H_2$ sorption data at 313 K and 1 bar on SIFSIX-dps-Cu.

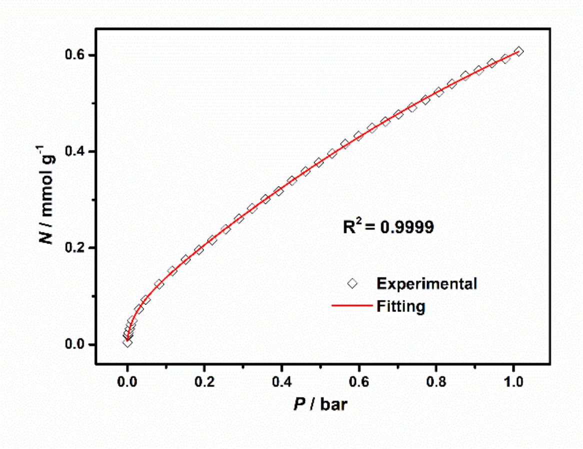


**Supplementary Figure 21.** DSLF fitting of the $CO_2$ sorption data at 298 K and 1 bar on SIFSIX-dps-Cu.

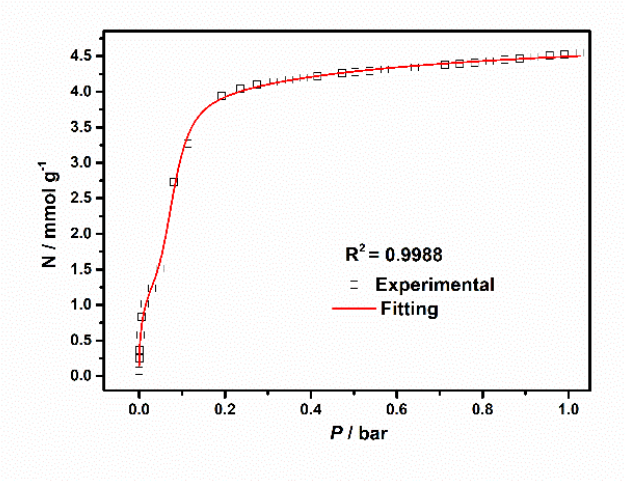


**Supplementary Figure 22.** DSLF fitting of the $C_2H_2$ sorption data at 273 K and 1 bar on GeFSIX-dps-Cu.

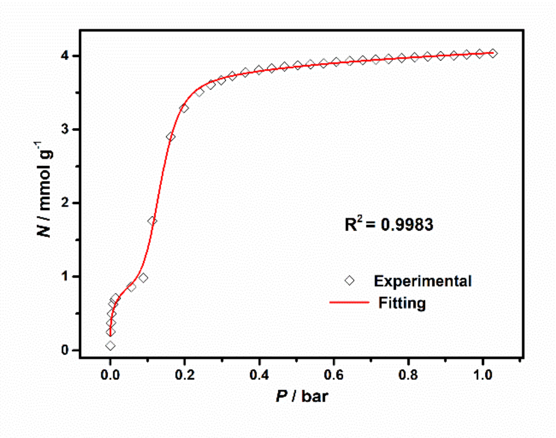


**Supplementary Figure 23.** DSLF fitting of the $C_2H_2$ sorption data at 298 K and 1 bar on GeFSIX-dps-Cu.

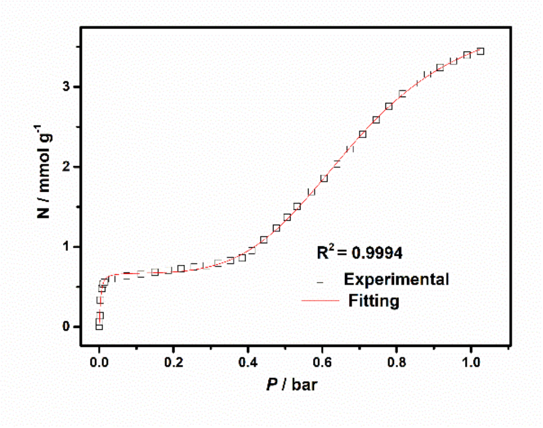


**Supplementary Figure 24.** DSLF fitting of the $C_2H_2$ sorption data at 323 K and 1 bar on GeFSIX-dps-Cu.

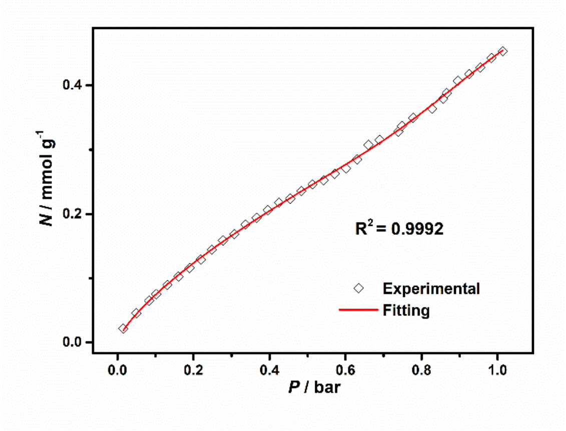


**Supplementary Figure 25.** DSLF fitting of the $CO_2$ sorption data at 298 K and 1 bar on GeFSIX-dps-Cu.

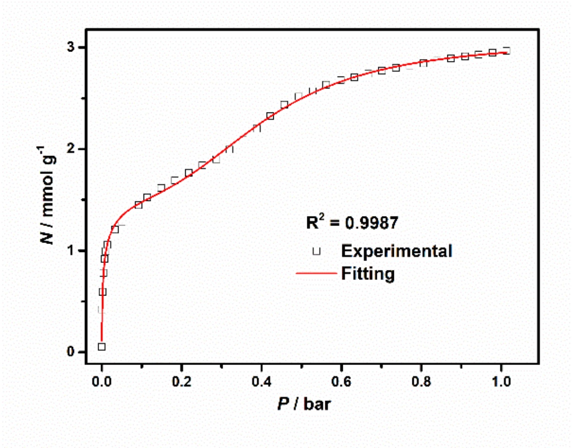


**Supplementary Figure 26.** DSLF fitting of the $C_2H_2$ sorption data at 273 K and 1 bar on NbOFFIVE-dps-Cu.

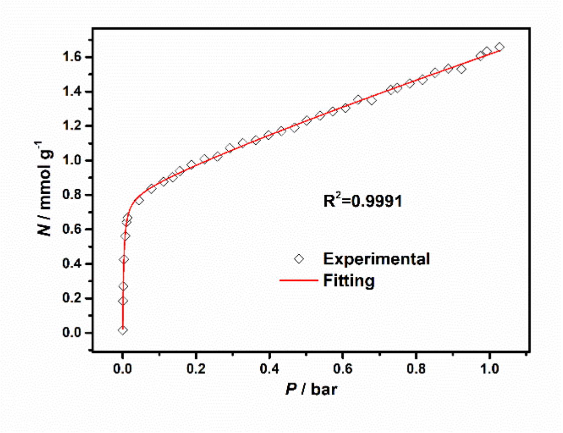


**Supplementary Figure 27.** DSLF fitting of the $C_2H_2$ sorption data at 298 K and 1 bar on NbOFFIVE-dps-Cu.

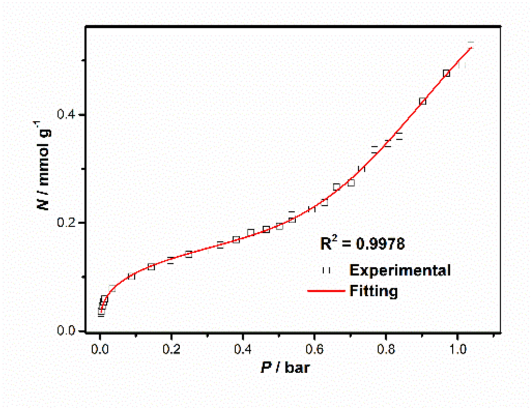


**Supplementary Figure 28.** DSLF fitting of the $C_2H_2$ sorption data at 323 K and 1 bar on NbOFFIVE-dps-Cu.

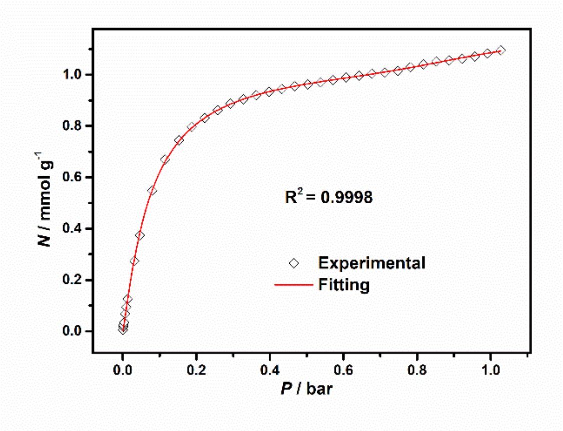


**Supplementary Figure 29.** DSLF fitting of the $CO_2$ sorption data at 298 K and 1 bar on NbOFFIVE-dps-Cu.

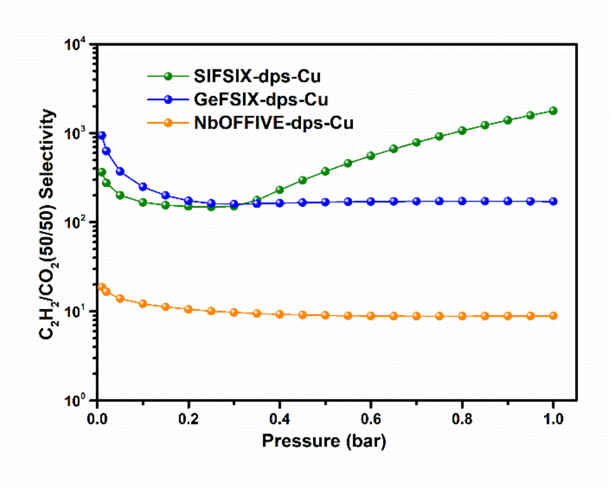


**Supplementary Figure 30.** IAST selectivity for $C_2H_2/CO_2$ (50/50) mixtures at 298 K.

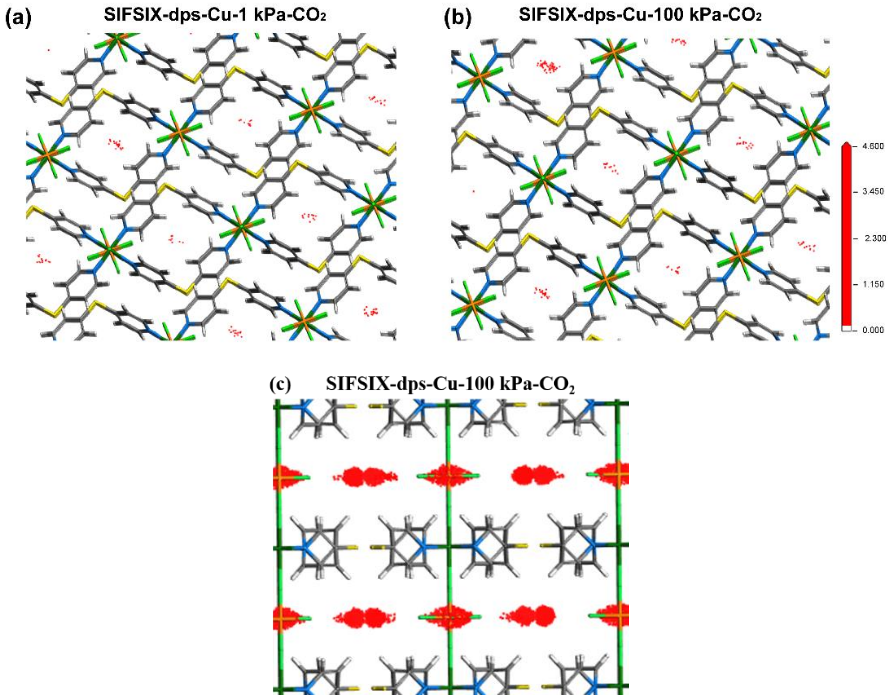


**Supplementary Figure 31.** The density distribution of $CO_2$ in SIFSIX-dps-Cu at (a) 1 kPa, (b) 100 kPa, viewed along the $(CuSiF_6)_\infty$ chains, and corresponding side view for (c) $C_2H_2$ at 100 kPa.

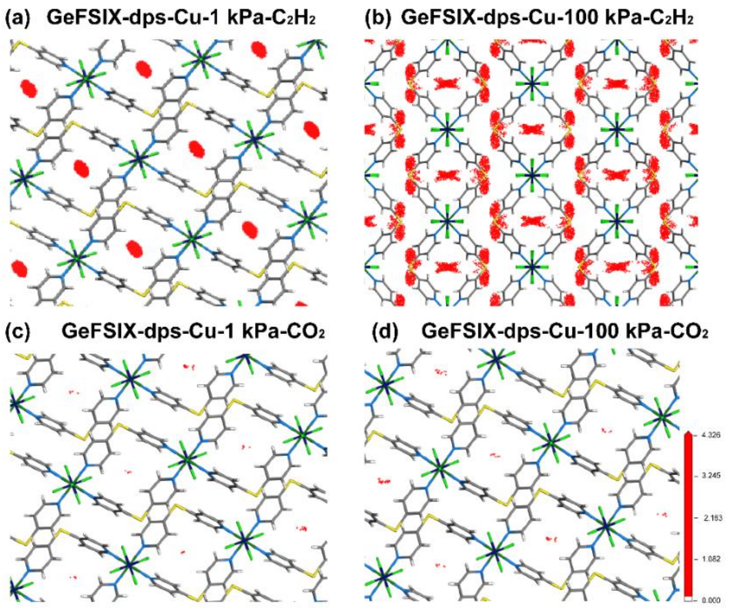


**Supplementary Figure 32.** The density distribution of $C_2H_2$ on GeFSIX-dps-Cu at (a) 1 kPa and (b) 100 kPa; $CO_2$ on GeFSIX-dps-Cu at (c) 1 kPa and (d) 100 kPa, viewed along the $(CuGeF_6)_\infty$ chains.

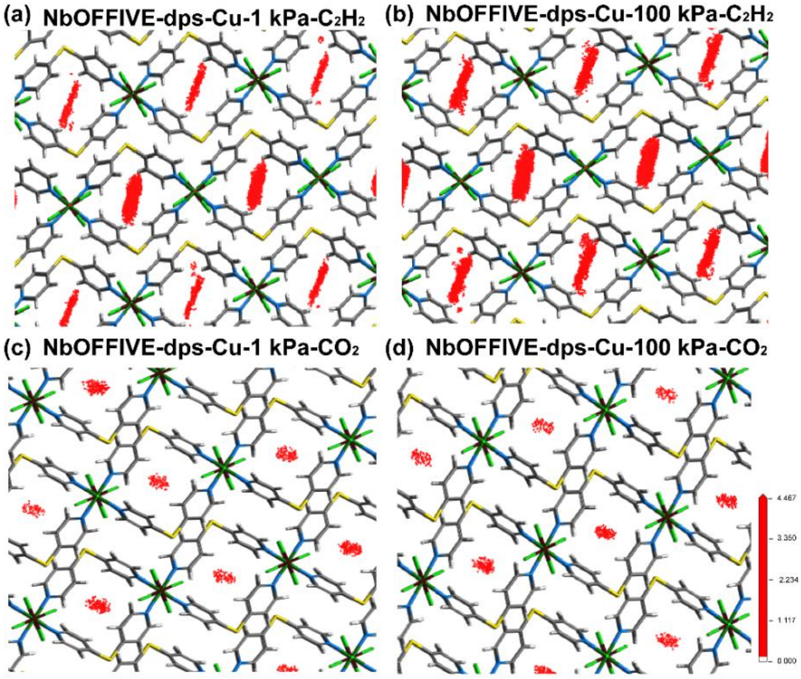


**Supplementary Figure 33.** The density distribution of $C_2H_2$ on NbOFFIVE-dps-Cu at (a) 1 kPa and (b) 100 kPa; $CO_2$ on NbOFFIVE-dps-Cu at (c) 1 kPa and (d) 100 kPa, viewed along the $(CuNbOF_5)_\infty$ chains.

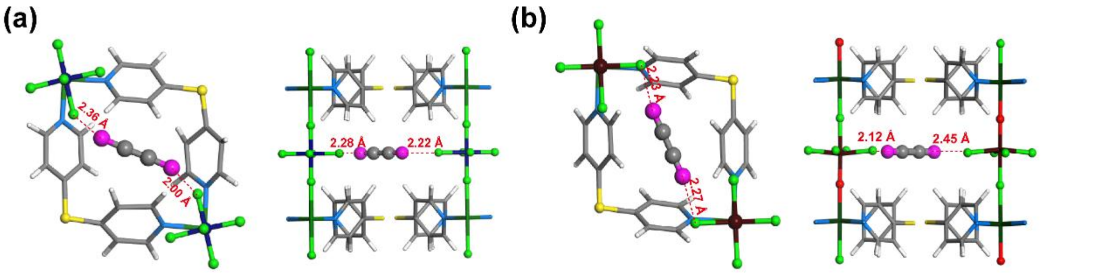


**Supplementary Figure 34.** DFT-D calculated $C_2H_2$ binding mode in (a) GeFSIX-dps-Cu and (b) NbOFFIVE-dps-Cu at sites I and II, respectively.

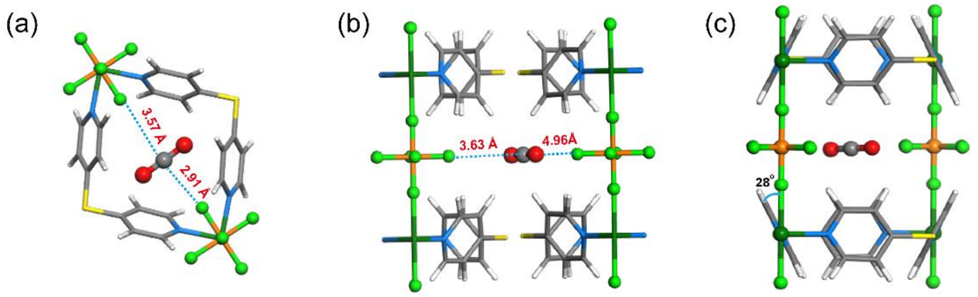


**Supplementary Figure 35.** The DFT-D calculated $CO_2$ binding mode (a) at site I and (b and c, viewed from different perspectives) at site II in SIFSIX-ds-Cu.

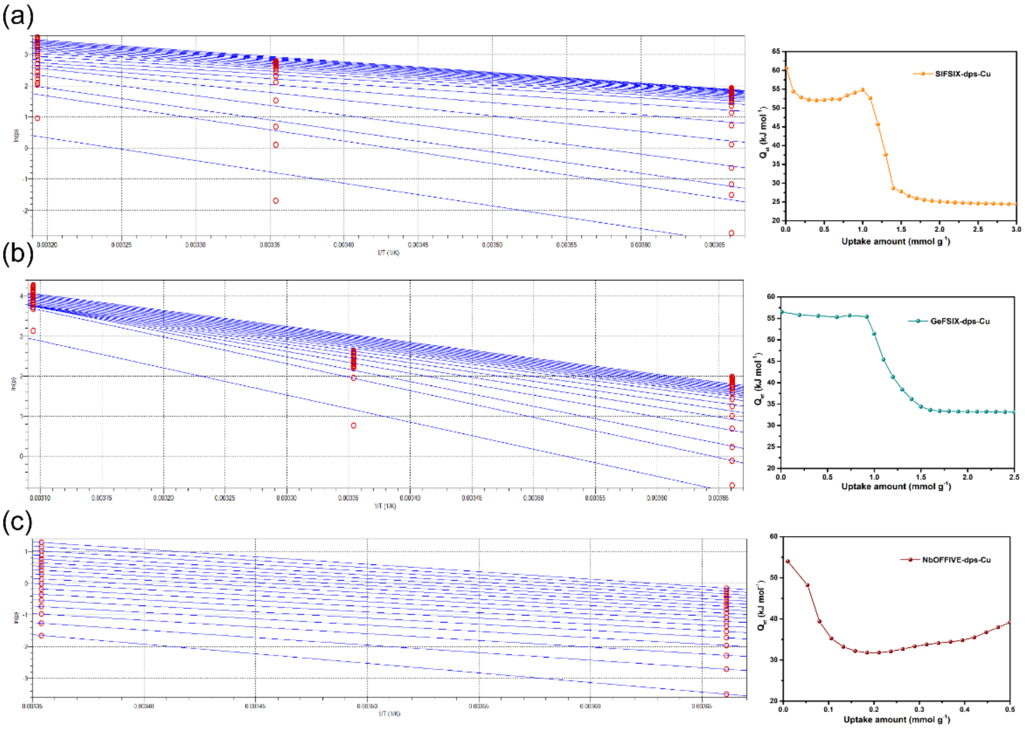


**Supplementary Figure 36**. Clausius-Clapeyron plots and corresponding $Q_{st}$ of (a) SIFSIX-dps-Cu, (b) GeFSIX-dps-Cu, and (c) NbOFFIVE-dps-Cu.

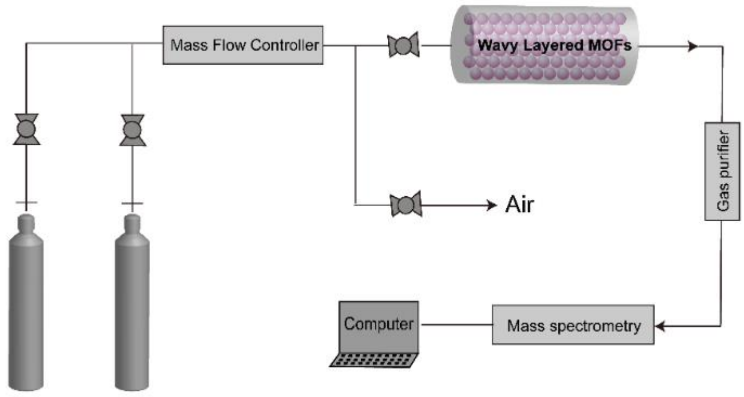


**Supplementary Figure 37.** Breakthrough experiments apparatus.

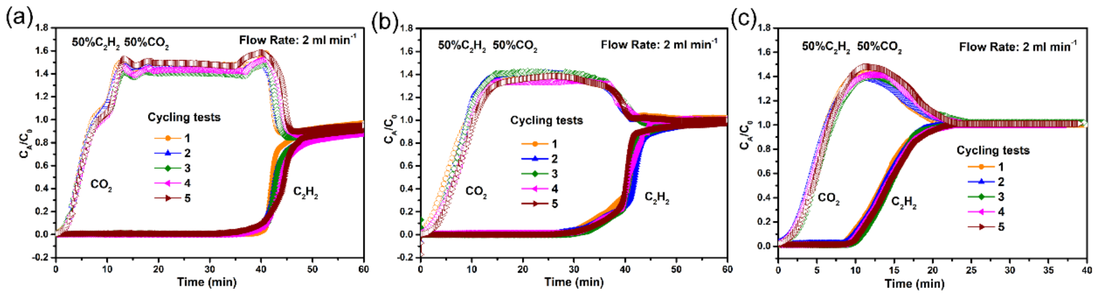


**Supplementary Figure 38.** Breakthrough experiment of equimolar $C_2H_2/CO_2$ mixture on (a) SIFSIX-dps-Cu, (b) GeFSIX-dps-Cu, and (c) NbOFFIVE-dps-Cu.

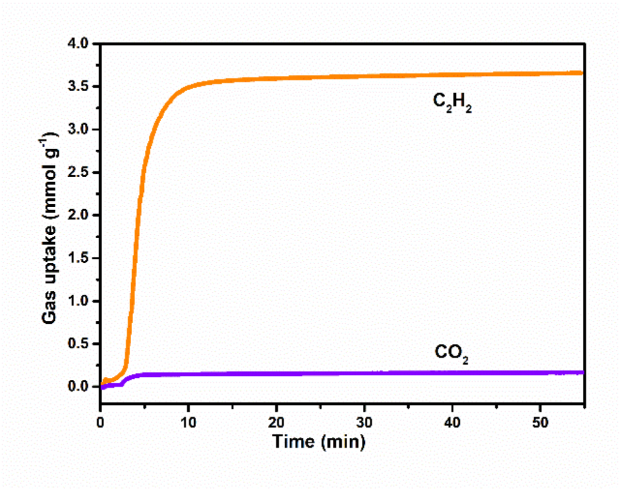


**Supplementary Figure 39.** $C_2H_2$ and $CO_2$ adsorption kinetic curves of SIFSIX-dps-Cu at 0.5 bar and 298 K.

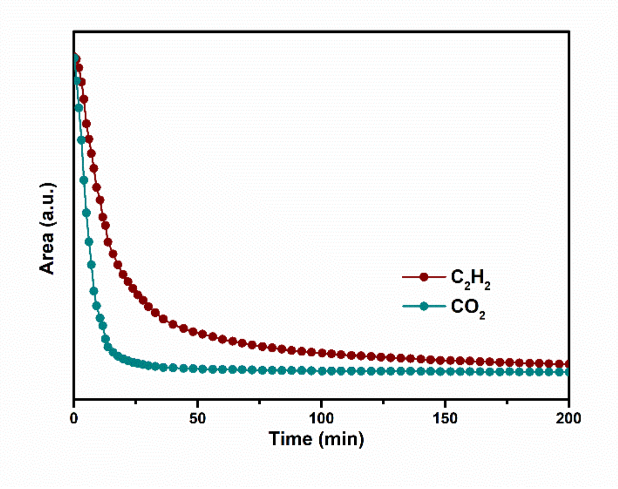


**Supplementary Figure 40.** The signals of desorbed gases from NbOFFIVE-dps-Cu in the regeneration process of breakthrough separation.

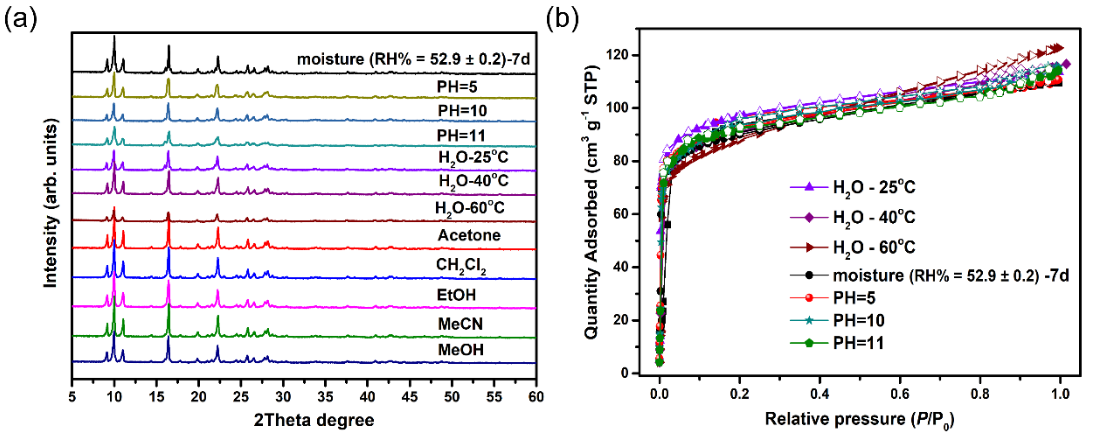


**Supplementary Figure 41.** Stability study of SIFSIX-dps-Cu. (a) Powder X-ray diffraction patterns upon various conditions, and (b) corresponding $CO_2$ sorption isotherms at 195 K. Note: The humidity is controlled by saturated $Mg(NO_3)_2$ solution at 298 K and 1.0 bar, with a relative humidity of RH 53%.

**Supplementary Table 1.** Crystal data and structure refinements

| Complex | SIFSIX-dps-Cu [39] | GeFSIX-dps-Cu | NbOFFIVE-dps-Cu |
| --- | --- | --- | --- |
| Formula. | $C_{20}H_{28}CuF_6SiN_4O_6S_2$ | $C_{20}H_{28}CuF_6GeN_4O_6S_2$ | $C_{20}H_{16}CuF_6N_4NbS_2$ |
| F.W. | 690.21 | 734.71 | 788.06 |
| Crystal size (mm) | 0.18×0.17×0.12 | 0.18×0.17×0.12 | 0.12×0.11×0.08 |
| Crystal system | Orthorhombic | Orthorhombic | Orthorhombic |

| | | | |
|---|---|---|---|
| Space group | *Ibam* | *Ibam* | *Ibam* |
| *a* /Å | 19.040 (4) | 19.037 (3) | 18.976(2) |
| *b* /Å | 9.9063 (18) | 9.8925 (14) | 9.8977(7) |
| *c* /Å | 15.835 (3) | 16.138 (3) | 16.2554(12) |
| $\alpha$/° | 90 | 90 | 90 |
| $\beta$/° | 90 | 90 | 90 |
| $\gamma$/° | 90 | 90 | 90 |
| volume/Å3 | 2986.7 (10) | 3039.2 (8) | 3053.1 (5) |
| Z | 4 | 4 | 4 |
| $D_c$ (g cm$^{-3}$) | 1.535 | 1.606 | 1.714 |
| $\mu$ / mm$^{-1}$ | 5.53 | 5.89 | 1.29 |
| $\theta$ range (°) | 6.3-53.9 | 4.0-53.8 | 2.5-32.4 |
| Ref. meas./indep. | 7810/1362 | 11964/1442 | 10122/1445 |
| Obs. ref.[I > 2σ (I)] | 1062 | 912 | 1242 |
| $R_{int}$ | 0.078 | 0.078 | 0.035 |
| F000 | 1412 | 1484 | 1596 |
| $R_1$ [$I \geq 2\sigma(I)$] [a] | 0.075 | 0.057 | 0.079 |
| $\omega R_2$(all data)[b] | 0.258 | 0.208 | 0.337 |
| GOF | 1.08 | 1.03 | 1.20 |
| Δρ(max, min) (e Å$^{-3}$) | 1.05, -0.65 | 0.67, -1.01 | 1.70, -2.19 |
| CCDC | 1907864 | 2060207 | 2060208 |

Notes: All the three structures here were obtained with same methodology at the same time. During our preparation on this work, the structures of GeFSIX-dps-Cu (CCDC: 1986967) and ZUL-220 (CCDC: 1974873) reported in Ref. 40 and 42, respectively, were found to show same components with those of the latter two. To facilitate the structure comparisons in our study, we here published our data for completeness.

**Supplementary Table 2.** Modelling study and refinement parameters of $C_2H_2$-loaded MOFs.

| Complex | $C_2H_2$-SIFSIX-dps-Cu | $C_2H_2$-GeFSIX-dps-Cu | $C_2H_2$-NbOFFIVE-dps-Cu |
|---|---|---|---|

| Formula. | $C_{104}H_{88}Cu_4F_{24}Si_4N_{16}S_8$ | $C_{104}H_{88}Cu_4F_{24}Ge_4N_{16}S_8$ | $C_{52}H_{44}Cu_2F_{10}N_8Nb_2O_2S_4$ |
|---|---|---|---|
| Space group | *Ibam* | *Ibam* | *P*2/*n* |
| F.W. | 2640.9 | 2857.3 | 1393.1 |
| *a* /Å | 21.2004 | 21.2588 | 9.8912 |
| *b* /Å | 9.595 | 9.7073 | 8.3000 |
| *c* /Å | 14.0064 | 14.2375 | 16.8612 |
| *β*/º | 90 | 90 | 97.7621 |
| volume/Å$^3$ | 2849.4 | 2904.4 | 1371.6 |
| $D_c$ (g cm$^{-3}$) | 1.539 | 1.643 | 1.691 |
| $R_{wp}$ | 0.0281 | 0.0205 | 0.0352 |
| $R_p$ | 0.0144 | 0.0153 | 0.0179 |
| $X^2$ | 1.05 | 1.09 | 1.05 |

**Supplementary Table 3**. Coordinates of the non-hydrogen atoms in simulated model of $C_2H_2$-loaded SIFSIX-dps-Cu.

| Atom | *x* | *y* | *z* | Atom | *x* | *y* | *z* |
|---|---|---|---|---|---|---|---|
| Cu1 | 0.50014 | -0.00237 | 0.25012 | C25 | -0.11454 | 0.63554 | 0.68301 |
| Cu2 | -0.00008 | 0.50025 | 0.74848 | C26 | 0.44881 | 0.24996 | 0.31221 |
| Cu3 | 0.49746 | -0.00231 | 0.74641 | C27 | 0.39212 | 0.36001 | 0.31525 |
| Cu4 | -0.00169 | 0.45012 | 0.24331 | C28 | 0.34025 | 0.35858 | 0.25252 |
| S1 | 0.70644 | 0.49847 | 0.24555 | C29 | 0.33909 | 0.24745 | 0.19006 |
| S2 | 0.28975 | 0.49791 | 0.25636 | C30 | 0.38559 | 0.14001 | 0.19247 |
| S3 | -0.20972 | -0.00419 | 0.75376 | C31 | 0.05866 | 0.24485 | 0.81664 |
| S4 | 0.20847 | -0.0033 | 0.74654 | C32 | 0.10858 | 0.13547 | 0.81027 |
| S5 | 0.28985 | 0.49793 | 0.74174 | C33 | 0.15636 | 0.13878 | 0.74731 |
| S6 | 0.70528 | 0.49649 | 0.75706 | C34 | 0.16121 | 0.24006 | 0.68899 |
| S7 | 0.20915 | -0.0035 | 0.25202 | C35 | 0.11636 | 0.35777 | 0.68205 |
| S8 | -0.20888 | -0.00331 | 0.24589 | C36 | 0.5594 | 0.74623 | 0.30911 |
| Si1 | 0.49607 | -0.00613 | -0.00118 | C37 | 0.60334 | 0.63808 | 0.30545 |
| Si2 | -0.00141 | 0.49319 | 0.49906 | C38 | 0.65204 | 0.63874 | 0.24337 |
| Si3 | 0.00154 | 0.49455 | -0.0008 | C39 | 0.65904 | 0.75125 | 0.18849 |
| Si4 | 0.50237 | -0.00694 | 0.49801 | C40 | 0.61442 | 0.85929 | 0.18423 |
| F1 | 0.49752 | -0.00332 | 0.11895 | C41 | 0.43928 | 0.74526 | 0.80874 |
| F2 | -0.00069 | 0.49492 | 0.61917 | C42 | 0.39674 | 0.63564 | 0.80692 |
| F3 | 0.00075 | 0.49456 | 0.87885 | C43 | 0.34855 | 0.63656 | 0.74113 |
| F4 | 0.50263 | -0.00381 | 0.37775 | C44 | 0.33912 | 0.75202 | 0.68313 |
| F5 | 0.49755 | -0.00334 | 0.87872 | C45 | 0.38361 | 0.85938 | 0.68505 |

| | | | | | | | |
|---|---|---|---|---|---|---|---|
| F6 | -0.00121 | 0.49666 | 0.37374 | C46 | -0.06022 | 0.24403 | 0.31059 |
| F7 | 0.00092 | 0.49461 | 0.11928 | C47 | -0.1029 | 0.13462 | 0.30974 |
| F8 | 0.50066 | -0.00359 | 0.61905 | C48 | -0.15664 | 0.13121 | 0.24544 |
| F9 | 0.45437 | 0.14221 | -0.00123 | C49 | -0.16024 | 0.24885 | 0.18472 |
| F10 | 0.56486 | 1.08053 | -0.00116 | C50 | -0.11343 | 0.35112 | 0.18434 |
| F11 | -0.04541 | 0.64657 | 0.50125 | C51 | 0.55766 | 0.25011 | 0.81319 |
| F12 | 0.06762 | 0.57909 | 0.49918 | C52 | 0.60443 | 0.36121 | 0.81222 |
| F13 | 0.53493 | 0.84159 | -0.00094 | C53 | 0.65167 | 0.3594 | 0.75296 |
| F14 | 0.42751 | -0.0943 | -0.00124 | C54 | 0.65323 | 0.24223 | 0.69443 |
| F15 | 0.03364 | 0.34542 | 0.49746 | C55 | 0.61529 | 0.13996 | 0.68988 |
| F16 | -0.06973 | 0.40391 | 0.49943 | C56 | 0.05863 | 0.74814 | 0.31227 |
| F17 | 0.04279 | 0.6416 | -0.00049 | C57 | 0.10103 | 0.8582 | 0.31244 |
| F18 | -0.06565 | 0.57334 | -0.0005 | C58 | 0.15434 | 0.85121 | 0.25554 |
| F19 | 0.5433 | 0.1415 | 0.49881 | C59 | 0.16041 | 0.74388 | 0.18944 |
| F20 | 0.43271 | 1.07701 | 0.49887 | C60 | 0.11666 | 0.63767 | 0.18876 |
| F21 | -0.03121 | 0.33846 | -0.00047 | C61 | 0.0593 | 0.24363 | 0.18803 |
| F22 | 0.07046 | 0.40438 | -0.00096 | C62 | 0.10202 | 0.13448 | 0.1885 |
| F23 | 0.46278 | 0.84044 | 0.49907 | C63 | 0.1526 | 0.13625 | 0.25161 |
| F24 | 0.57007 | -0.09708 | 0.49901 | C64 | 0.16012 | 0.24899 | 0.31454 |
| N1 | 0.56667 | 0.14022 | 0.25001 | C65 | 0.11535 | 0.35631 | 0.31198 |
| N2 | 0.06324 | 0.64742 | 0.74283 | C66 | 0.55948 | 0.74646 | 0.68855 |
| N3 | 0.43312 | 0.85664 | 0.25101 | C67 | 0.60217 | 0.63727 | 0.69004 |
| N4 | -0.06656 | 0.35245 | 0.7505 | C68 | 0.65199 | 0.63876 | 0.75448 |
| N5 | -0.06575 | 0.64631 | 0.75075 | C69 | 0.65556 | 0.75232 | 0.81121 |
| N6 | 0.43858 | 0.14666 | 0.25105 | C70 | 0.61439 | 0.85926 | 0.81369 |
| N7 | 0.06646 | 0.35205 | 0.74704 | C71 | -0.05931 | 0.74734 | 0.18523 |
| N8 | 0.56517 | 0.85409 | 0.24695 | C72 | -0.10164 | 0.85739 | 0.18483 |
| N9 | 0.43309 | 0.85387 | 0.74733 | C73 | -0.15273 | 0.85667 | 0.24722 |
| N10 | -0.06223 | 0.35343 | 0.24555 | C74 | -0.16445 | 0.74339 | 0.30409 |
| N11 | 0.56486 | 0.14342 | 0.75046 | C75 | -0.11612 | 0.63718 | 0.30949 |
| N12 | 0.06563 | 0.64074 | 0.24998 | C76 | 0.44048 | 0.24916 | 0.68418 |
| N13 | 0.06559 | 0.35132 | 0.25028 | C77 | 0.39803 | 0.35896 | 0.68286 |
| N14 | 0.56532 | 0.85436 | 0.75064 | C78 | 0.34648 | 0.35815 | 0.7443 |
| N15 | -0.0645 | 0.64121 | 0.24565 | C79 | 0.33821 | 0.24597 | 0.80606 |
| N16 | 0.43454 | 0.14555 | 0.7489 | C80 | 0.38285 | 0.13899 | 0.8076 |
| C1 | 0.55883 | 0.25004 | 0.18939 | C81 | 0.27677 | 1.6156 | 1.99749 |
| C2 | 0.60015 | 0.35996 | 0.18062 | C82 | 0.31292 | 1.71404 | 1.99874 |
| C3 | 0.65781 | 0.35226 | 0.24333 | C83 | 0.48028 | 1.43512 | 1.99891 |
| C4 | 0.66121 | 0.24894 | 0.31121 | C84 | 0.50438 | 1.54868 | 1.99892 |
| C5 | 0.61026 | 0.14542 | 0.30743 | C85 | 0.18326 | 0.75786 | 0.49914 |
| C6 | 0.06015 | 0.75012 | 0.68556 | C86 | 0.21553 | 0.86148 | 0.49903 |
| C7 | 0.10262 | 0.85945 | 0.68456 | C87 | 0.5184 | 0.43497 | 0.49919 |
| C8 | 0.15394 | 0.85248 | 0.74015 | C88 | 0.49399 | 0.54659 | 0.50012 |
| C9 | 0.16514 | 0.7327 | 0.81026 | C89 | 0.98401 | -0.06405 | 0.4651 |

| | | | | | | | |
|---|---|---|---|---|---|---|---|
| C10 | 0.11014 | 0.63482 | 0.81064 | C90 | 1.00823 | 0.04952 | 0.49885 |
| C11 | 0.44029 | 0.74575 | 0.18305 | C91 | 1.31695 | 0.24167 | 0.49888 |
| C12 | 0.39783 | 0.63481 | 0.19642 | C92 | 1.28222 | 0.34278 | 0.499 |
| C13 | 0.34639 | 0.63751 | 0.25485 | C93 | 1.01634 | -0.06606 | 0.99947 |
| C14 | 0.33917 | 0.75157 | 0.3151 | C94 | 0.99545 | 0.04889 | 0.99012 |
| C15 | 0.38527 | 0.85905 | 0.31374 | C95 | 1.22336 | 0.12578 | 0.99874 |
| C16 | -0.06094 | 0.24444 | 0.68884 | C96 | 1.18684 | 0.22133 | 0.99927 |
| C17 | -0.10385 | 0.13612 | 0.69054 | C97 | 0.68193 | 1.24785 | 0.99888 |
| C18 | -0.152 | 0.13509 | 0.75746 | C98 | 0.7163 | 1.34808 | 0.99883 |
| C19 | -0.16896 | 0.25001 | 0.81474 | C99 | 0.77343 | 1.10754 | 0.49902 |
| C20 | -0.11378 | 0.35064 | 0.81115 | C100 | 0.81006 | 1.20624 | 0.49647 |
| C21 | -0.06041 | 0.74221 | 0.81301 | C101 | 0.72866 | 0.58347 | 0.49662 |
| C22 | -0.10111 | 0.85565 | 0.81222 | C102 | 0.69182 | 0.69028 | 0.4989 |
| C23 | -0.15301 | 0.85877 | 0.75599 | C103 | 0.81647 | 0.74336 | 0.99887 |
| C24 | -0.16447 | 0.74556 | 0.69001 | C104 | 0.78476 | 0.84479 | 0.99769 |

**Supplementary Table 4**. Coordinates of the non-hydrogen atoms in simulated model of $C_2H_2$-loaded GeFSIX-dps-Cu.

| Atom | *x* | *y* | *z* | Atom | *x* | *y* | *z* |
|---|---|---|---|---|---|---|---|
| Cu1 | 0.50014 | -0.00237 | 0.25012 | C25 | -0.11454 | 0.63554 | 0.68301 |
| Cu2 | -0.00008 | 0.50025 | 0.74848 | C26 | 0.44881 | 0.24996 | 0.31221 |
| Cu3 | 0.49746 | -0.00231 | 0.74641 | C27 | 0.39212 | 0.36001 | 0.31525 |
| Cu4 | -0.00169 | 0.45012 | 0.24331 | C28 | 0.34025 | 0.35858 | 0.25252 |
| S1 | 0.70644 | 0.49847 | 0.24555 | C29 | 0.33909 | 0.24745 | 0.19006 |
| S2 | 0.28975 | 0.49791 | 0.25636 | C30 | 0.38559 | 0.14001 | 0.19247 |
| S3 | -0.20972 | -0.00419 | 0.75376 | C31 | 0.05866 | 0.24485 | 0.81664 |
| S4 | 0.20847 | -0.0033 | 0.74654 | C32 | 0.10858 | 0.13547 | 0.81027 |
| S5 | 0.28985 | 0.49793 | 0.74174 | C33 | 0.15636 | 0.13878 | 0.74731 |
| S6 | 0.70528 | 0.49649 | 0.75706 | C34 | 0.16121 | 0.24006 | 0.68899 |
| S7 | 0.20915 | -0.0035 | 0.25202 | C35 | 0.11636 | 0.35777 | 0.68205 |
| S8 | -0.20888 | -0.00331 | 0.24589 | C36 | 0.5594 | 0.74623 | 0.30911 |
| Ge1 | 0.49712 | -0.00605 | -0.00126 | C37 | 0.60334 | 0.63808 | 0.30545 |
| Ge2 | -0.00153 | 0.49331 | 0.50017 | C38 | 0.65204 | 0.63874 | 0.24337 |
| Ge3 | 0.00147 | 0.49512 | -0.00073 | C39 | 0.65904 | 0.75125 | 0.18849 |

| | | | | | | | |
|---|---|---|---|---|---|---|---|
| Ge4 | 0.50301 | -0.00712 | 0.49793 | C40 | 0.61442 | 0.85929 | 0.18423 |
| F1 | 0.49752 | -0.00332 | 0.11895 | C41 | 0.43928 | 0.74526 | 0.80874 |
| F2 | -0.00069 | 0.49492 | 0.61917 | C42 | 0.39674 | 0.63564 | 0.80692 |
| F3 | 0.00075 | 0.49456 | 0.87885 | C43 | 0.34855 | 0.63656 | 0.74113 |
| F4 | 0.50263 | -0.00381 | 0.37775 | C44 | 0.33912 | 0.75202 | 0.68313 |
| F5 | 0.49755 | -0.00334 | 0.87872 | C45 | 0.38361 | 0.85938 | 0.68505 |
| F6 | -0.00121 | 0.49666 | 0.37374 | C46 | -0.06022 | 0.24403 | 0.31059 |
| F7 | 0.00092 | 0.49461 | 0.11928 | C47 | -0.1029 | 0.13462 | 0.30974 |
| F8 | 0.50066 | -0.00359 | 0.61905 | C48 | -0.15664 | 0.13121 | 0.24544 |
| F9 | 0.45437 | 0.14221 | -0.00123 | C49 | -0.16024 | 0.24885 | 0.18472 |
| F10 | 0.56486 | 1.08053 | -0.00116 | C50 | -0.11343 | 0.35112 | 0.18434 |
| F11 | -0.04541 | 0.64657 | 0.50125 | C51 | 0.55766 | 0.25011 | 0.81319 |
| F12 | 0.06762 | 0.57909 | 0.49918 | C52 | 0.60443 | 0.36121 | 0.81222 |
| F13 | 0.53493 | 0.84159 | -0.00094 | C53 | 0.65167 | 0.3594 | 0.75296 |
| F14 | 0.42751 | -0.0943 | -0.00124 | C54 | 0.65323 | 0.24223 | 0.69443 |
| F15 | 0.03364 | 0.34542 | 0.49746 | C55 | 0.61529 | 0.13996 | 0.68988 |
| F16 | -0.06973 | 0.40391 | 0.49943 | C56 | 0.05863 | 0.74814 | 0.31227 |
| F17 | 0.04279 | 0.6416 | -0.00049 | C57 | 0.10103 | 0.8582 | 0.31244 |
| F18 | -0.06565 | 0.57334 | -0.0005 | C58 | 0.15434 | 0.85121 | 0.25554 |
| F19 | 0.5433 | 0.1415 | 0.49881 | C59 | 0.16041 | 0.74388 | 0.18944 |
| F20 | 0.43271 | 1.07701 | 0.49887 | C60 | 0.11666 | 0.63767 | 0.18876 |
| F21 | -0.03121 | 0.33846 | -0.00047 | C61 | 0.0593 | 0.24363 | 0.18803 |
| F22 | 0.07046 | 0.40438 | -0.00096 | C62 | 0.10202 | 0.13448 | 0.1885 |
| F23 | 0.46278 | 0.84044 | 0.49907 | C63 | 0.1526 | 0.13625 | 0.25161 |
| F24 | 0.57007 | -0.09708 | 0.49901 | C64 | 0.16012 | 0.24899 | 0.31454 |
| N1 | 0.56667 | 0.14022 | 0.25001 | C65 | 0.11535 | 0.35631 | 0.31198 |
| N2 | 0.06324 | 0.64742 | 0.74283 | C66 | 0.55948 | 0.74646 | 0.68855 |
| N3 | 0.43312 | 0.85664 | 0.25101 | C67 | 0.60217 | 0.63727 | 0.69004 |
| N4 | -0.06656 | 0.35245 | 0.7505 | C68 | 0.65199 | 0.63876 | 0.75448 |

| | | | | | | | |
|---|---|---|---|---|---|---|---|
| N5 | -0.06575 | 0.64631 | 0.75075 | C69 | 0.65556 | 0.75232 | 0.81121 |
| N6 | 0.43858 | 0.14666 | 0.25105 | C70 | 0.61439 | 0.85926 | 0.81369 |
| N7 | 0.06646 | 0.35205 | 0.74704 | C71 | -0.05931 | 0.74734 | 0.18523 |
| N8 | 0.56517 | 0.85409 | 0.24695 | C72 | -0.10164 | 0.85739 | 0.18483 |
| N9 | 0.43309 | 0.85387 | 0.74733 | C73 | -0.15273 | 0.85667 | 0.24722 |
| N10 | -0.06223 | 0.35343 | 0.24555 | C74 | -0.16445 | 0.74339 | 0.30409 |
| N11 | 0.56486 | 0.14342 | 0.75046 | C75 | -0.11612 | 0.63718 | 0.30949 |
| N12 | 0.06563 | 0.64074 | 0.24998 | C76 | 0.44048 | 0.24916 | 0.68418 |
| N13 | 0.06559 | 0.35132 | 0.25028 | C77 | 0.39803 | 0.35896 | 0.68286 |
| N14 | 0.56532 | 0.85436 | 0.75064 | C78 | 0.34648 | 0.35815 | 0.7443 |
| N15 | -0.0645 | 0.64121 | 0.24565 | C79 | 0.33821 | 0.24597 | 0.80606 |
| N16 | 0.43454 | 0.14555 | 0.7489 | C80 | 0.38285 | 0.13899 | 0.8076 |
| C1 | 0.55883 | 0.25004 | 0.18939 | C81 | 0.27677 | 1.6156 | 1.99749 |
| C2 | 0.60015 | 0.35996 | 0.18062 | C82 | 0.31292 | 1.71404 | 1.99874 |
| C3 | 0.65781 | 0.35226 | 0.24333 | C83 | 0.48028 | 1.43512 | 1.99891 |
| C4 | 0.66121 | 0.24894 | 0.31121 | C84 | 0.50438 | 1.54868 | 1.99892 |
| C5 | 0.61026 | 0.14542 | 0.30743 | C85 | 0.18326 | 0.75786 | 0.49914 |
| C6 | 0.06015 | 0.75012 | 0.68556 | C86 | 0.21553 | 0.86148 | 0.49903 |
| C7 | 0.10262 | 0.85945 | 0.68456 | C87 | 0.5184 | 0.43497 | 0.49919 |
| C8 | 0.15394 | 0.85248 | 0.74015 | C88 | 0.49399 | 0.54659 | 0.50012 |
| C9 | 0.16514 | 0.7327 | 0.81026 | C89 | 0.98401 | -0.06405 | 0.4651 |
| C10 | 0.11014 | 0.63482 | 0.81064 | C90 | 1.00823 | 0.04952 | 0.49885 |
| C11 | 0.44029 | 0.74575 | 0.18305 | C91 | 1.31695 | 0.24167 | 0.49888 |
| C12 | 0.39783 | 0.63481 | 0.19642 | C92 | 1.28222 | 0.34278 | 0.499 |
| C13 | 0.34639 | 0.63751 | 0.25485 | C93 | 1.01634 | -0.06606 | 0.99947 |
| C14 | 0.33917 | 0.75157 | 0.3151 | C94 | 0.99545 | 0.04889 | 0.99012 |
| C15 | 0.38527 | 0.85905 | 0.31374 | C95 | 1.22336 | 0.12578 | 0.99874 |
| C16 | -0.06094 | 0.24444 | 0.68884 | C96 | 1.18684 | 0.22133 | 0.99927 |
| C17 | -0.10385 | 0.13612 | 0.69054 | C97 | 0.68193 | 1.24785 | 0.99888 |
| C18 | -0.152 | 0.13509 | 0.75746 | C98 | 0.7163 | 1.34808 | 0.99883 |
| C19 | -0.16896 | 0.25001 | 0.81474 | C99 | 0.77343 | 1.10754 | 0.49902 |
| C20 | -0.11378 | 0.35064 | 0.81115 | C100 | 0.81006 | 1.20624 | 0.49647 |
| C21 | -0.06041 | 0.74221 | 0.81301 | C101 | 0.72866 | 0.58347 | 0.49662 |
| C22 | -0.10111 | 0.85565 | 0.81222 | C102 | 0.69182 | 0.69028 | 0.4989 |
| C23 | -0.15301 | 0.85877 | 0.75599 | C103 | 0.81647 | 0.74336 | 0.99887 |
| C24 | -0.16447 | 0.74556 | 0.69001 | C104 | 0.78476 | 0.84479 | 0.99769 |

**Supplementary Table 5**. Coordinates of the non-hydrogen atoms in simulated model of $C_2H_2$-loaded NbOFFIVE-dps-Cu.

| Atom | *x* | *y* | *z* | Atom | *x* | *y* | *z* |
|---|---|---|---|---|---|---|---|
| Cu1 | 1 | 0.19358 | 0 | C10 | 0.16592 | -0.21998 | -0.37134 |
| Cu2 | 0.5 | -0.32326 | -0.5 | C11 | 0.12294 | 0.07599 | -0.14355 |
| S1 | 0.05058 | -0.34566 | -0.23798 | C12 | 0.2209 | 0.06499 | -0.19706 |
| S2 | 0.44309 | 0.16688 | -0.26076 | C13 | 0.32285 | 0.17998 | -0.18578 |
| Nb1 | 1 | 0.67643 | 0 | C14 | 0.33484 | 0.29997 | -0.13459 |
| Nb2 | 0.5 | -0.83331 | -0.5 | C15 | 0.23289 | 0.29997 | -0.07699 |
| F1 | 0.82481 | 0.71092 | 0.03933 | C16 | 0.59672 | 0.16798 | -0.19826 |
| F2 | 1.10248 | 0.69992 | 0.10843 | C17 | 0.6317 | 0.05299 | -0.13256 |
| F3 | 0.38082 | -0.82291 | -0.5962 | C18 | 0.73965 | 0.06699 | -0.08229 |
| F4 | 0.33684 | -0.82291 | -0.44638 | C19 | 0.80562 | 0.29297 | -0.14512 |
| F5 | 1 | 0.9209 | 0 | C20 | 0.70567 | 0.27997 | -0.20068 |
| F6 | 0.5 | -0.58694 | -0.5 | C21 | 0.29736 | 0.16672 | 0.32179 |
| N1 | -0.34384 | -0.33796 | -0.41383 | C22 | 0.42121 | 0.16835 | 0.33599 |
| N2 | 0.35783 | -0.31797 | -0.42217 | C23 | 0.05445 | 0.69746 | 0.17313 |
| N3 | 0.12494 | 0.19298 | -0.08617 | C24 | 0.17858 | 0.70157 | 0.1823 |
| N4 | 0.83191 | 0.18498 | -0.08532 | O1 | 1 | 0.45795 | 0 |
| C1 | -0.23989 | -0.44295 | -0.41854 | O2 | 0.5 | -1.05888 | -0.5 |
| C2 | -0.11595 | -0.45295 | -0.36696 | | | | |
| C3 | -0.10495 | -0.33496 | -0.30858 | | | | |
| C4 | -0.19491 | -0.22198 | -0.29705 | | | | |
| C5 | -0.32485 | -0.22698 | -0.35073 | | | | |
| C6 | 0.16892 | -0.33496 | -0.30706 | | | | |
| C7 | 0.28487 | -0.44395 | -0.31087 | | | | |
| C8 | 0.36483 | -0.42895 | -0.36459 | | | | |
| C9 | 0.25388 | -0.20998 | -0.42046 | | | | |

**Supplementary Table 6.** Summary of the adsorption uptakes and selectivities for $C_2H_2$ and $CO_2$ in various MOFs

| Sample | $C_2H_2$ (mmol $g^{-1}$) | $CO_2$ (mmol $g^{-1}$) | IAST (50/50) | Working Capacity (mmol $g^{-1}$) | $C_2H_2/CO_2$ Breakthrough time (min $g^{-1}$) | *Ref* |
|---|---|---|---|---|---|---|
| SIFSIX-dps-Cu | 4.57 | 0.61 | 1786.6 | 2.48 | 53 | This work |
| GeFSIX-dps-Cu | 4.04 | 0.45 | 171.9 | 2.36 | 50 | This work |
| NbOFFIVE-dp-Cu | 1.65 | 1.10 | 9.0 | 0.83 | 14 | This work |
| JNU-1 | 2.75 | 2.25 | 5.0 | 2.84 | 34.1 | 1 |
| ATC-Cu | 5.01 | 4.02 | 53.6 | 6.9 | 133 | 2 |
| UTSA-300a | 3.08 | 0.15 | >$10^4$ | 0.77 | 12 | 3 |
| NTU-65 | 3.36 | 0.10 | - | 2.08 | 125 | 4 |
| CPL-1-$NH_2$ | 1.84 | 0.21 | 119 | 1.38 | 28 | 5 |
| SIFSIX-3-Ni | 3.3 | 2.7 | 7.7 [a] | 2.5 | 75 | 6 |
| TIFSIX-2-Cu-i | 4.1 | 4.3 | 6.5 [a] | - | 97 | 6 |
| UTSA-74a | 4.82 | 2.85 | 9 | - | - | 7 |
| UTSA-222a | 3.81 [b] | 1.91 [b] | 2.0 [b] | - | - | 8 |
| SNNU-45 | 5.98 | 4.35 | 4.5 | 3.5 | 79 | 9 |
| FJU-90a | 8.04 | 4.60 | 4.3 | 1.87 | 22 | 10 |
| FJU-6-TATB | 4.91 [b] | 2.59 [b] | 3.1 [b] | 0.62 | 34 | 11 |

[a] Selectivity calculated from IAST for a $C_2H_2/CO_2$ (2:1) mixture;

[b] 296 K;

**Supplementary Table 7.** The DSLF fitting parameters for $C_2H_2$ and $CO_2$ in three isoreticular MOFs.

| Adsorbents | Adsorbates | $N_1^{max}$ (mmol g$^{-1}$) | $b_1$ (bar$^{-1}$) | $1/n_1$ | $N_2^{max}$ (mmol g$^{-1}$) | $b_2$ (bar$^{-1}$) | $1/n_2$ |
|---|---|---|---|---|---|---|---|
| SIFSIX-Cu-dps | $C_2H_2$ (273 K) | 2.61 | 4.07E-12 | 10.8 | 3.56 | 1.86 | 0.342 |
| SIFSIX-Cu-dps | $C_2H_2$ (298 K) | 3.34 | 1.18 | 0.367 | 2.73 | 1.08E-14 | 17.9 |
| SIFSIX-Cu-dps | $C_2H_2$ (313 K) | 2.25 | 48.9 | 2.72 | 1.03 | 7.81 | 0.662 |
| SIFSIX-Cu-dps | $CO_2$ (298 K) | 0.985 | 0.652 | 1.41 | 0.313 | 2.15 | 0.575 |
| GeFSIX-Cu-dps | $C_2H_2$ (273 K) | 3.26 | 3.40 | 0.488 | 1.97 | 7.93E-4 | 4.49 |
| GeFSIX-Cu-dps | $C_2H_2$ (298 K) | 3.56 | 0.779 | 0.299 | 2.48 | 5.44E-4 | 5.48 |
| GeFSIX-Cu-dps | $C_2H_2$ (323 K) | 0.675 | 2.69E-3 | 1.43 | 3.28 | 5.19 | 4.40 |
| GeFSIX-Cu-dps | $CO_2$ (298 K) | 0.0831 | 1.99 | 7.98 | 5.81 | 0.0726 | 0.751 |
| NbOFFIVE-Cu-dps | $C_2H_2$ (273 K) | 1.84 | 12.9 | 0.522 | 1.33 | 13.7 | 2.97 |
| NbOFFIVE-Cu-dps | $C_2H_2$ (298 K) | 0.805 | 3.47E-2 | 1.02 | 7.26 | 0.126 | 1.00 |
| NbOFFIVE-Cu-dps | $C_2H_2$ (323 K) | 3.17 | 0.0744 | 0.328 | 0.531 | 1.09 | 4.96 |
| NbOFFIVE-Cu-dps | $CO_2$ (298 K) | 0.108 | 1.62 | 6.90 | 1.07 | 20.2 | 1.17 |

**Supplementary References**


1. Zeng, H. *et al.* Induced fit of $C_2H_2$ in a flexible MOF through cooperative action of open metal sites. *Angew. Chem. Int. Ed.* **58**, 8515–8519 (2019).

2. Niu, Z. *et al.* A MOF-based ultra-strong acetylene nano-trap for highly efficient $C_2H_2/CO_2$ separation. *Angew. Chem. Int. Ed.* **60**, 5283–5288 (2021).

3. Lin, R. B. *et al.* Optimized separation of acetylene from carbon dioxide and ethylene in a microporous material. *J. Am. Chem. Soc.* **139**, 8022–8028 (2017).

4. Dong, Q. *et al.* Tuning gate-opening of a flexible metal–organic framework for ternary gas sieving separation. *Angew. Chem. Int. Ed.* **59**, 22756–22762 (2020).

5. Yang, L. Z. *et al.* Adsorption site selective occupation strategy within a metal–organic framework for highly efficient sieving acetylene from carbon dioxide. *Angew. Chem. Int. Ed.* **60**, 4570 – 4574 (2021).

6. Hamon, L. *et al.* Co-adsorption and separation of $CO_2$−$CH_4$ mixtures in the highly flexible MIL-53(Cr) MOF. *J. Am. Chem. Soc.* **131**, 17490-17499 (2009).

7. Chen, K. *et al.* Benchmark $C_2H_2/CO_2$ and $CO_2/C_2H_2$ separation by two closely related hybrid ultramicroporous materials. *Chem* **1**, 753–765 (2016).

8. Luo, F. *et al.* UTSA-74: A MOF-74 isomer with two accessible binding sites per metal center for highly selective gas separation. *J. Am. Chem. Soc.* **138**, 5678–5684 (2016).

9. Ma, J. X. *et al.* Microporous lanthanide metal-organic framework constructed from lanthanide metalloligand for selective separation of $C_2H_2/CO_2$ and $C_2H_2/CH_4$ at room temperature. *Inorg. Chem.* **56**, 7145–7150 (2017).

10. Li, Y. P. *et al.* Ultramicroporous building units as a path to bi-microporous metal-organic frameworks with high acetylene storage and separation performance. *Angew. Chem. Int. Ed.* **58**, 13590 –13595 (2019).

11. Fan, W. *et al.* Optimizing multivariate metal–organic frameworks for efficient $C_2H_2/CO_2$ separation. *J. Am. Chem. Soc.* **142***,* 8728-8737 (2020).
